# Characterization of the *doublesex* gene within the *Culex pipiens* complex suggests regulatory plasticity at the base of the mosquito sex determination cascade

**DOI:** 10.1186/s12862-015-0386-1

**Published:** 2015-06-11

**Authors:** Dana C. Price, Andrea Egizi, Dina M. Fonseca

**Affiliations:** Department of Entomology, Rutgers University, 178 Jones Avenue, New Brunswick, NJ 08901 USA; Graduate Program in Ecology and Evolution, Rutgers University, New Brunswick, NJ USA

**Keywords:** *doublesex*, mab-3, *Culex quinquefasciatus*, *Culex pipiens*, Sterile insect technique, Vector biology, Sex determination

## Abstract

**Background:**

The *doublesex* gene controls somatic sexual differentiation of many metazoan species, including the malaria mosquito *Anopheles gambiae* and the dengue and yellow fever vector *Aedes aegypti* (Diptera: Culicidae). As in other studied dipteran *dsx* homologs, the gene maintains functionality via evolutionarily conserved protein domains and sex-specific alternative splicing. The upstream factors that regulate splicing of *dsx* and the manner in which they do so however remain variable even among closely related organisms. As the induction of sex ratio biases is a central mode of action in many emerging molecular insecticides, it is imperative to elucidate as much of the sex determination pathway as possible in the mosquito disease vectors.

**Results:**

Here we report the full-length gene sequence of the *doublesex* gene in *Culex quinquefasciatus* (*Cxqdsx*) and its male and female-specific isoforms. *Cxqdsx* maintains characteristics possibly derived in the Culicinae and present in the *Aedes aegypti dsx* gene (*Aeadsx*) such as gain of exon 3b and the presence of Rbp1 *cis*-regulatory binding sites, and also retains presumably ancestral attributes present in *Anopheles gambiae* such as maintenance of a singular female-specific exon 5. Unlike in *Aedes aegypti*, we find no evidence for intron gain in the female transcript(s), yet recover a second female isoform generated via selection of an alternate splice donor. Utilizing next-gen sequence (NGS) data, we complete the *Aeadsx* gene model and identify a putative core promoter region in both *Aeadsx* and *Cxqdsx.* Also utilizing NGS data, we construct a full-length gene sequence for the *dsx* homolog of the northern house mosquito *Culex pipiens* form pipiens (*Cxpipdsx*). Analysis of peptide evolutionary rates between *Cxqdsx* and *Cxpipdsx* (both members of the *Culex pipiens* complex) shows the male-specific portion of the transcript to have evolved rapidly with respect to female-specific and common regions.

**Conclusions:**

As in other studied insects, *doublesex* maintains sex-specific splicing and conserved *doublesex*/mab-3 domains in the mosquitoes *Culex quinquefasciatus* and *Cx. pipiens*. The *cis*-regulated splicing of *Cxqdsx* does not appear to follow either currently described mosquito model (for *An. gambiae* and *Ae. aegypti*); each of the three mosquito genera exhibit evidence of unique cis-regulatory mechanisms. The male-specific *dsx* terminus exhibits rapid peptide evolutionary rates, even among closely related sibling species.

**Electronic supplementary material:**

The online version of this article (doi:10.1186/s12862-015-0386-1) contains supplementary material, which is available to authorized users.

## Background

The manifestation of distinct sexes is fundamentally conserved among most metazoans. However, the development of sex-specific somatic and gonadal tissues and neuronal processes (e.g. behaviors) is governed by a variety of factors both environmental and genetic, and often varying widely between and within taxa [1–3]. Most animals direct sex-specific cell fate by function of the *Doublesex*/Mab-3 Related Transcription factor (DMRT) family of zinc-finger proteins [4, 5] and the genes they regulate. Within the insects, this process involves a genetic cascade first elucidated in the model fly *Drosophila melanogaster* [6] whereby a primary signal triggers sex-specific splicing of one or more regulatory factors which subsequently bind pre-mRNA of the conserved DMRT “major switch” gene, *doublesex*, and direct its sex-specific splicing, thus initiating development of male or female forms [7].

Although there are many diverse primary signals that initiate the cascade (e.g. X:A ratio, M-factors, W/Y chromosomes; see [1]), *dsx* appears to be conserved as the major switch at the base of the cascade [8, 9]. In many insects the male and female-specific splicing of *dsx* is directed by the upstream regulator *transformer*, a serine/arginine rich (SR) protein which itself is transcribed in a sex-specific manner, as well as the constitutively expressed *transformer-2* [10, 11]. The resultant TRA/TRA2 peptide complex binds the *dsx* mRNA at the *dsx* repeat element (dsxRE), facilitated by the purine-rich enhancer (PRE) element [12, 13], and directs sex-specific splicing of *dsx* mRNA for translation into male (DSX^M^) or female (DSX^F^) peptides. In *Drosophila*, an additional SR splicing enhancer component, RBP1, binds to target sites in the splice acceptor preceding the female-specific exon and is essential for efficient splicing of female *dsx* pre-mRNA [14]. The downstream targets of insect *dsx* are not well elucidated, however 58 optimal binding sites and associated nearest genes have been identified for *D. melanogaster Dmdsx* [15]*.* The red flour beetle *Tribolium castaneum Tcdsx* has been implicated in oocyte development including Vitellogenins and their associated receptors [16], while Lepidopteran *dsx* has been shown to influence expression of pheromone-binding proteins and *hexamerin* storage proteins [17].

Orthologs of the *dsx* gene have currently been identified in seven orders of insects ranging from the primitive *Pediculus humanus* (human body louse) to several genera of Hymenoptera, however a functional *transformer* homolog has not always been recovered in these genomes (see [8] for summary) leading to speculation that some lineages have recruited alternate or additional upstream regulators for *dsx* [18]. For example, TRA/TRA-2 mediated splicing of *dsx* has been shown in the Brachyceran flies *Ceratitis capitata* [19], *Musca domestica* [20] and *Lucilia cuprina* [21] yet transformer appears lost in the Nematoceran flies including mosquitoes [8].

Despite varying primary signals and upstream regulatory mechanisms, male and female-specific DSX peptides of various Diptera including *Anastrepha* [22], *Ceratitis* [23] and *Musca* [24] effected partial masculinization and feminization of genetically female and male *D. melanogaster*, respectively, when expressed ectopically. This evolutionary conservation is due in part to the retention of two functional protein domains essential for peptide oligomerization: an atypical zinc-finger DNA-binding domain found in multiple members of the DMRT superfamily (DBD/OD1) and an oligomerization domain (OD2) unique to *dsx* [25]. The DBD/OD1 domain functions to form a dimeric DNA-binding unit that maintains 92 % sequence similarity between Dipteran (*D. melanogaster*) and Lepidopteran (*Bombyx mori*) taxa while completely conserving the critical cysteine and histidine residues [26]. The OD2 domain is likely responsible for sex-specific splicing activation or repression of downstream factors [25], and is modified by sex-specific splicing to maintain both common and male/female-specific portions; the common portion exhibits a greater degree of conservation within and among insect taxa than the C-terminal sex-specific portion [18, 26].

**Fig. 1:**
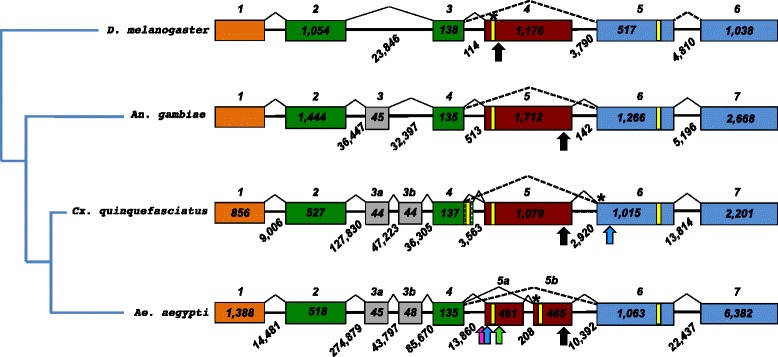
Organization and splicing of *D. melanogaster*, *An. gambiae*, *Cx. quinquefasciatus* and *Ae. aegypti doublesex* genes. Homologous exons (colored boxes) are aligned vertically, with numbers placed above. Numbers within boxes represent the exon size, while those on a diagonal below represent intron size. Yellow bars (not placed to scale) represent stop codons. Solid splice guides follow the female-specific spliceform, while dashed guides represent splicing in the male-specific form. Common exons are shown in green, the female-specific exon 5 in dark red, and male-specific (UTR in female) exons in blue. The green/white stippled box adjacent to exon 4 denotes the extension of the reading frame to the alternate splice donor. Black arrows denote TRA/TRA2 binding sites, purple arrows denote putative TRA2-ISS motif clusters, blue arrows signal RBP1 type-b motif clusters and green arrows signal Nasonia-like NvTRA binding site location. Asterisks are placed above weak splice acceptors

Orthologs of *dsx* have been recovered from the mosquitoes *Anopheles gambiae* (*Angdsx* [27]) and *Aedes aegypti* (*Aeadsx* [18]). Both genes show sex-specific splicing and contain multiple copies of TRA/TRA2 *cis*-regulatory elements including dsxREs and purine-rich enhancers, however they differ in several evolutionary aspects. The *Angdsx* gene (Fig. 1) spans an 85 kb region of chromosome 2R and is composed of seven exons, of which the first four code for 5’ UTR and a common non-sex specific region of the protein. Exon 5 is female-specific (i.e. is spliced out of the male mRNA) and contains an in-frame stop codon terminating the female peptide. Exon 6 contains male-specific coding sequence with termination codon and 3’ UTR, and exon 7 contains only 3’UTR. Exons 6 and 7 are present in transcripts of both sexes but are transcribed entirely as UTR in the female isoform. Female-specific splicing of *Angdsx* and the retention of exon 5 relies on activation of a 5’ splice donor (Fig. 1; see [28] for alternative splicing mechanism review) of the downstream intron 5 following binding of a TRA/TRA2 complex to dsxREs which facilitates recruitment of the spliceosomal machinery. This is in contrast to *doublesex* genes of *D. melanogaster, Bactrocera tryoni, M. domestica, M. scalaris* and *C. capitata*, which splice the female-specific isoform after activation of a weak (due to the presence of purines in the polypyrimidine tract) 3’ splice acceptor upstream of the female-specific exon to facilitate its inclusion. This is evidenced by the location of the dsxRE in *Angdsx* being at the 3’ end of the female-specific exon as opposed to the 5’ end as in *Dmdsx*.

The current *Aeadsx* gene model [18] (Fig. 1) spans 450 kb of genomic DNA of supercontig 1.370 and is composed of eight known exons, although nine are likely. Unlike other sequenced Dipteran *dsx* genes, *Aeadsx* was found to produce two female-specific isoforms by exon skipping, encoding peptides with alternative C-termini via inclusion of both exons 5a and 5b, or 5b alone. Additionally, analysis of *cis*-acting elements in *Aeadsx* revealed a cluster of TRA-2-ISS and RBP1 elements upstream of exon 5a, and Dipteran dsxRE binding sites and PRE elements present only in exon 5b (Fig. 1). Several instances of a motif strongly resembling a potential dsxRE element previously only recovered in the Hymenoptera (*NvdsxRE*, [11]) were found within exon and intron 5a. Unlike *An. gambiae* (and similar to *Drosophila*) *Aeadsx* possesses a weak splice acceptor upstream of exon 5b that is activated to splice both female isoforms. Salvemini et al. [18] hypothesize that regulatory mechanisms governing the sex-specific splicing of the gene in *Ae. aegypti* are different than in other Diptera including *An. gambiae*, and that the two female-specific exons were each under the control of a different splicing regulator: A female-specific TRA-like protein acts in females as a splicing activator of exon 5b via dsxRE and PRE elements, while a splice repressor acts on 5a (included by default splicing) in some transcripts. In the males, a male-specific factor may act to repress inclusion of exon 5a via TRA-2-ISS and *NvdsxRE* elements, while exon 5b is excluded due to lack of female-specific TRA.

Cho et al. [29] proposed that default female-specific *dsx* splicing by selective repression of the male isoform (i.e. by the *feminizer* gene in *A. mellifera* [30] and the recently discovered piRNA precursor *Fem* in *B. mori* [31]) is ancestral to holometabolous insects based on its conservation in taxa as phylogenetically distant as *A. mellifera* and *B. mori*, and that Diptera possess a derived splicing system where the male form is default and the female form must be ‘splice-activated’ by a TRA/TRA2-like factor. While this appears to be the case in *Anastrepha*, *Drosophila,* and *An. gambiae doublesex*, the data from Salvemini et al. [18] strongly suggest that the female spliceforms are default in *Ae. aegypti*; the “strong” exon 5a does not require TRA/TRA2 enhancement, and must be repressed by a male factor. Culicine mosquitoes (inclusive of the genera *Aedes* and *Culex*) determine sex at an autosomal locus [32], while Anopheline mosquitoes possess heteromorphic (XY) sex chromosomes [33]. The latter authors propose that this locus (the M-locus) may either act on intermediary factors or on the *dsx* gene itself (*transformer* appears to be either lost or extremely diverged in the mosquitoes [8], however *transformer2* is present) to suppress female-specific *dsx* splicing and generate the male form. Further, Salvemini et al. [18] posit that retention of the Hymenopteran-like *NvdsxRE* elements coupled with *Apis*-like splicing regulation (and a likely female-specific default splicing) could represent a stably maintained ancestral state in *Ae. aegypti* exclusive of the rest of known Dipteran *doublesex*. Recently, analysis of the red flour beetle *Tribolium castaneum* [34] revealed three female-specific and one male-specific *dsx* isoform, with male default splicing occurring via suppression of maternally transferred zygotic TRA protein (required to activate female-specific splicing) by a dominant male factor. This variation in the top-level regulation of *dsx* among Hymenoptera, Diptera, Lepidoptera, and Coleoptera via upstream factors is in agreement with the theory of Wilkins [35] stating that the cascade has evolved in reverse order, with the final double-switch gene (*doublesex*) remaining relatively conserved as additional elements are added and/or neofunctionalization occurs at the upper regulatory levels. As sex determination is critical to insect reproduction, deleterious mutations in *dsx* could therefore have strong effects on fitness and be selected against. Previous studies have shown the female-specific exon to be evolutionarily conserved [36–38], yet disagree on evolutionary rate comparisons of the common and male-specific portions of the transcript over longer evolutionary time frames. Hughes [38] found a much greater rate of non-synonymous substitutions within the male-specific region as compared to the common region, while Sobrinho Jr. and de Brito [39] found nearly equivalent levels of positive selection between the two.

As the production of genetic sexing mosquito strains and molecular methods that create male bias and/or elimination of the female sex are ideal strategies for sterile insect technique [40], it follows that a conserved sex regulator like *doublesex* (and *transformer*) would be optimal molecular targets for such control programs [41]. Elucidating the variable mechanisms by which *dsx* determines sexual fate in sequenced mosquito lineages is mandatory if progress is to be made towards a control strategy for the world’s deadliest animals. Here we provide full-length gene sequence, sex-specific splicing analyses, and regulatory analysis of the *doublesex* gene from the southern house mosquito *Culex quinquefasciatus* (herein *Cxqdsx*) via RT-PCR and Illumina transcriptome data. Additionally, to discern the strength and location of early evolutionary drivers on *doublesex* within the *Culex pipiens* complex, we conduct an evolutionary analysis using *Cxqdsx* and a newly constructed *dsx* transcript from *Culex pipiens* form pipiens (*Cxpipdsx*). These results provide a comparative platform with which to study sex determination in those mosquitoes with currently sequenced genomes (*An. gambiae* [42], *Ae. aegypti* [43] and *Cx. quinquefasciatus* [44]).

## Methods

**Fig. 2:**
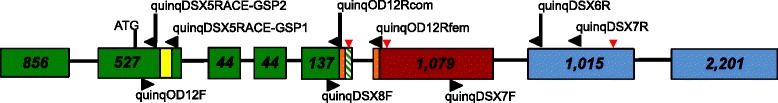
Location of *Cxqdsx* RT-PCR primers (not to scale). Common exons are shown in green, the female-specific exon 5 in dark red, and male-specific (UTR in female) exons in blue. The exon4ex extension is represented with a green/white hatched box. The DBD/OD1 domain is indicated with a yellow box and OD2 with an orange box. Red triangles denote stop codons

We used the conserved OD1 and OD2 peptide sequences of the *Aedes aegypti doublesex* gene [18] as a TBLASTN query to the *Cx. quinquefasciatus* genome assembly [44] and identified strong hits to both on genome supercontig 3.59. Further BLAST searches using the full peptide sequence of *Aeadsx* identified very weak local alignments to the supercontig representing putative female-specific (exon 5) and male specific/UTR (exon 6) coding sequence. A putative start codon in exon 2 was identified via homology with *Aeadsx*, and primers quinqOD12F, quinqOD12Rcom and quinqOD12Rfem (See Fig. 2 and Additional file 1: Table S1) were designed to amplify the putative 5’ end of the common and female specific transcripts, respectively, and primers quinqDSX8F, quinqDSX7F, quinqDSX6R and quinqDSX7R were designed to amplify the 3’ end of male and female specific transcripts.

*Culex quinquefasciatus* mosquitoes were obtained from a colony initiated in 2008 with egg rafts collected from Oahu, Hawaii, USA. Male and female total RNA was extracted separately from twenty adult mosquitoes of each sex using the Qiagen RNeasy Plus Universal Kit (Qiagen, Valencia CA) per manufacturer’s protocol. Prior to extraction, samples were placed in a 2 ml eppendorf tube containing a sterile steel bead + 800 μl Qiazol solution and homogenized for 1 min @ 20Hz on a Qiagen TissueLyser. Contaminant DNA was removed with the TURBO DNA-free DNA Removal Kit (Invitrogen, Carlsbad CA) and first-strand cDNA was generated using the Superscript First-Strand Synthesis System (Invitrogen) per manufacturer’s protocol and diluted to 50 μl in H2O. Four microliters of the cDNA was used in each 25 μl PCR reaction containing 12 μl H2O, 2.5 μl Qiagen Q-solution, 2.5 μl 10× PCR buffer, 0.5 μl dNTPs, 2.5 units AmpliTaq DNA Polymerase (Invitrogen) and 0.5 μl (200 μM final concentration) of each primer. Thermal cycling conditions were as follows: 1 min @ 95 °C, followed by 30 cycles x (30 s @ 94 °C, 30 s @ 50-54 °C primer-specific annealing, 60 s @ 68 °C [120 s for products > 1 kb]), 5 min @ 68 °C final extension.

To recover the complete 5’ end of the transcript, we performed 5’ RACE PCR using the FirstChoice RLM-RACE Kit (Invitrogen) per manufacturer’s protocol using internal gene-specific primers quinqDSX5RACE-GSP1 and quinqDSX5RACE-GSP2 placed adjacent to the OD1 domain. All RT-PCR and RACE-PCR amplicon products were visualized on a 1.5 % agarose gel in TAE buffer and gel-purified using the QIAquick Gel Extraction Kit (Qiagen) prior to cloning via the TOPO TA Cloning Kit (Invitrogen) and PCR-enrichment using the M13 forward/reverse primer pair per manufacturer’s protocol. PCR products were cleaned with ExoSap (Invitrogen) per manufacturer’s protocol, and cycle sequencing was performed by GENEWIZ (South Plainfield, NJ) using the M13 primer pair.

The 3’ end of *Cxqdsx* was predicted, and the entire gene sequence qualified by mapping the paired-end RNAseq data from NCBI SRA accession SRR991016 generated by Leal et al. [45] to *Cx. quinquefasciatus* supercontig 3.59 using the CLC Genomics Workbench (CLC Bio, Aarhus, Denmark) large-gap read mapper (nucleotide similarity score of 95 % over a 95 % read length fraction) and manually examining the output. This process was repeated using the *Cx. pipiens* f. pipiens paired-end RNAseq library generated by [46] (see Additional file 2 for details) and the *Cx. quinquefasciatus* reference generated above to create the full-length gene structure for *Cx. pipiens* f. pipiens *doublesex (Cxpipdsx)*. To extend the gene model for *Aeadsx*, we repeated this protocol yet again with the *Ae. aegypti* NCBI short-read paired-end libraries SRR924024 and SRR789758 and AaegL1.4 supercontig 1.370.

To assess the distribution of the consensus dsxRE (TRA/TRA2) and RBP1 type-b motifs (derived from those of *D. melanogaster*, *An. gambiae* and *Ae. aegypti*), we screened all transcript coding (CDS) sequences corresponding with the *Cx. quinquefasciatus* Cpip1.3 dataset from VectorBase for their presence. The degenerate motif was broken down into all possible constituents, and each was queried against the CDS dataset with BLASTn (e-val = 999, word_size = 13 [dsxRE] or 7 [RBP1b]). The output was parsed via custom Perl scripts, and transcripts containing six copies of the motif in a 224 bp (for the dsxRE; 546 bp for RBP1b) window were retained. The AhoPro software utility [47] was used to calculate the probability of observing the motif against a reference dataset of nucleotides randomly generated under a Bernoulli/0-order Markov model.

The synonymous substitutions per synonymous site and nonsynonymous substitutions per nonsynonymous site (Ks and Ka, respectively) and the Ka/Ks ratio were calculated in a pairwise comparison between *Cxqdsx* and *Cxpipdsx* using the KaKs Calculator v2.0 [48] under model averaging (MA). We re-calculated these values for each sliding 30 bp window while moving 3 bp (1 amino acid) downstream at a time. To examine base composition of splice acceptor sites, we retrieved 52,278 internal (i.e. exclusive of exon 1) exons with 16 nt of upstream sequence from the CpipJ 1.3 assembly (Vectorbase, [49]) and calculated the mean number of pyrimidines in the 12 nt preceding the 4 nt splice acceptor.

## Results and discussion

### Structure and splicing of *Cxqdsx*

**Fig. 3:**
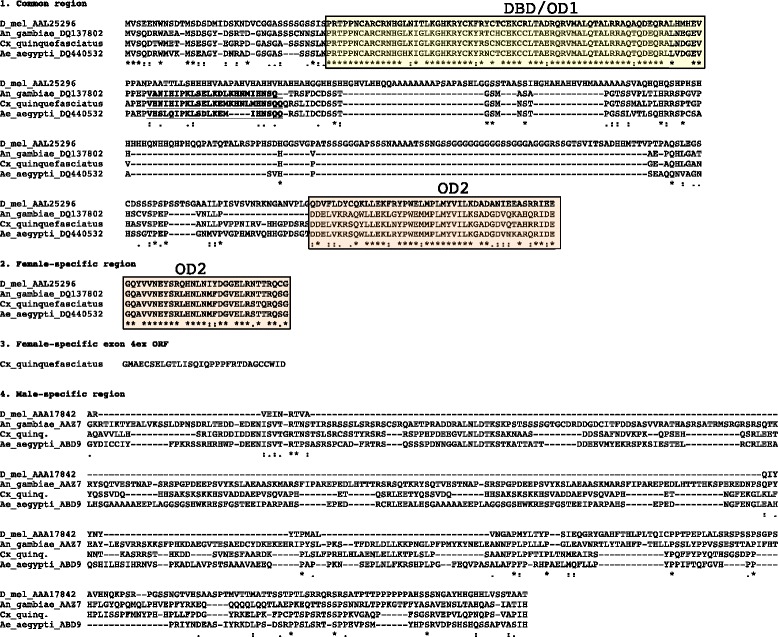
Peptide alignment of *D. melanogaster*, *An. gambiae*, *Cx. quinquefasciatus* and *Ae. aegypti doublesex* genes; divided into 1. Common region (present in both male and female peptide), 2. Female-specific region 1 (C-terminus of female-specific protein), 3. Female-specific peptide C-terminus generated by use of alternate exon 4 splice donor, and 4. Male-specific region (C-terminus of male-specific protein). NCBI identification numbers are appended to the sequence ID. The in-frame intronic sequence is in bold/underline. The DNA-binding oligomerization domain (DBD/OD1) is boxed in yellow, while the common and female-specific portions of OD2 are boxed in orange

**Fig. 4:**
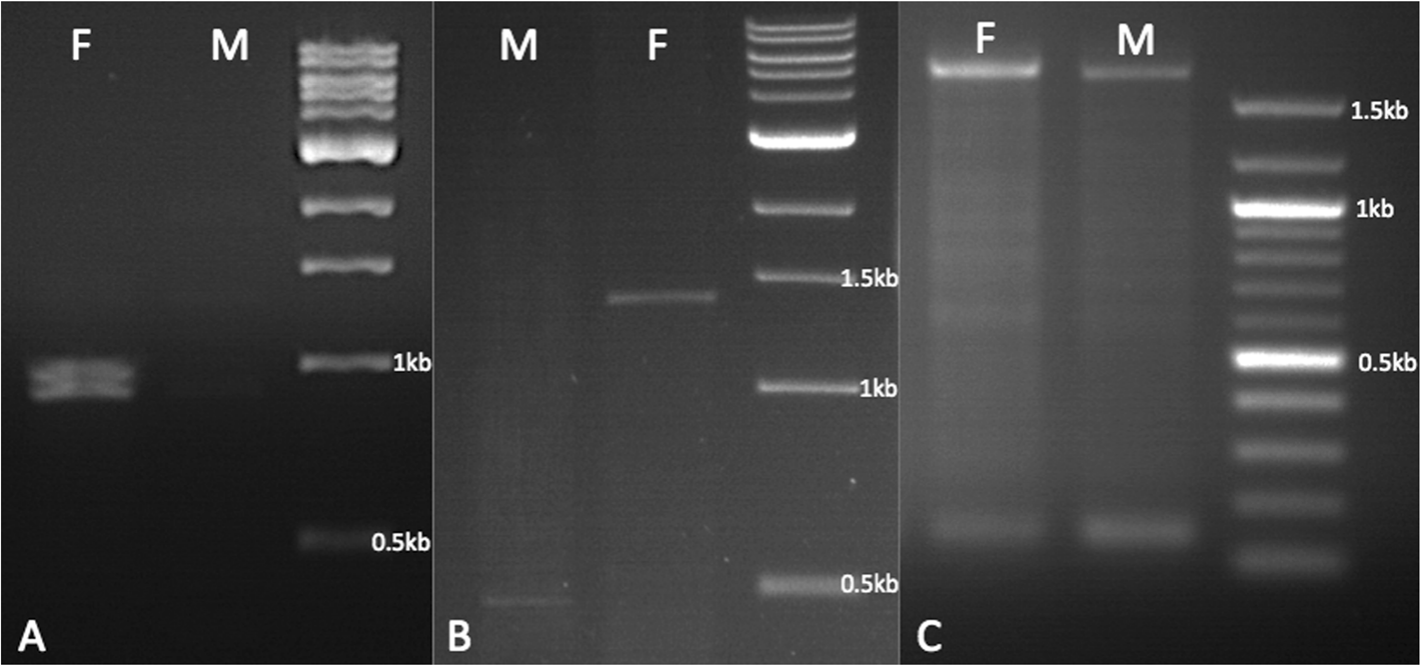
RT-PCR gel visualizations. **a**. RT-PCR products derived from primers quinqOD12F/quinqOD12Rfem used to amplify female (left) and male (right) cDNA. **b**. RT-PCR products derived from primers dsx8F/dsx6R used to amplify male (left) and female (right) cDNA. **c**. RT-PCR products derived from 5’RACE-PCR reaction after final amplification with primer DSX-5RACEGSP2 on female (left) and male (right) cDNA

TBLASTN identified strong alignments to both *Aeadsx* OD1 and OD2 domains on *Cx. quinquefasciatus* supercontig 3.59. Further homology searches via TBLASTN (not shown) identified putative local alignments to both the common (exons 2 and 4 of *Aeadsx*), female specific (exon 5) and male-specific (exon 6) CDS sequence on that same contig. The primer pair quinqOD12F/quinqOD12R (Fig. 2), designed to amplify the common regions of the OD1 and OD2 domains, produced a double-band in both male and female *Cx. quinquefasciatus* cDNA. Sequencing and genome alignment revealed this was due to the presence of a 75 bp (25 amino acid) alternatively spliced in-frame intronic sequence within exon 2 that was present in some transcripts but spliced out of others (Fig. 3). An equivalent 63 bp (21 amino acid) tract was reported from *Aeadsx* and a 72 bp (24 amino acid) tract reported in *Angdsx* [18], however this appears to be specific to the Culicidae and has not been reported from sequenced *dsx* transcripts in other taxa. The conservation and evolution of this splicing event within the mosquitoes is evidence of an as yet undetermined functional role. Both male and female N-termini of the *Cxqdsx* gene contained two small 45 bp exons homologous to exons 3a and 3b of *Aeadsx* (Fig. 1). The primer pair quinqOD12F/quinqOD12Rfem, designed to amplify the putative female-specific transcript by binding the 3’ end of the OD2 domain in exon 5, generated product only in female cDNA (Fig. 4a) thus confirming the sex-specific splicing of the mRNA and the location of the female-specific exon. By using a forward primer located downstream of the in-frame intron in exon 4 (DSX8F) and reverse primer within the putative male-specific/common exon 6 (DSX6R), we generated an amplicon spanning a ca. 1,079 bp exon (exon 5, Fig. 4b) specific to the female that was spliced to exon 6 after removal of 2.9 kb of intronic sequence (Fig. 1). We find no evidence for an alternative female spliceform involving a second female-specific exon as is present in *Aeadsx* [18]*,* indicating that the phenomenon is likely an intron gain in *Aedes* rather than a loss in *Anopheles*. The male RT-PCR product lacked this exon, and consisted of a smaller amplicon splicing exons 4 and 6 (Fig. 4b). Both males and females shared the C-terminal male-specific/common exon 6 (as UTR in the female) as in *Angdsx* and *Aeadsx*.

**Fig. 5:**
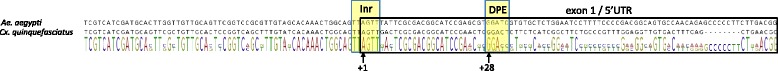
Pairwise nucleotide alignment of putative *doublesex* promoter regions of *Culex quinquefasciatus* (bottom) and *Aedes aegypti* (top). Initiator box (Inr) and downstream promoter element (DPE) are boxed in yellow. The transcription start site (TSS) at position +1 and DPE at position +28 are marked. Exon 1 (spliced as UTR in both male and female mRNA) is outlined in black. The sequence logo plot below the alignment illustrates conservation

5’ RACE-PCR, after final amplification with primer DSX-5RACEGSP2, produced an identical ca. 1400 bp amplicon from both male and female cDNA (Fig. 4c) that extends exon 2 of the transcript 451 bp upstream of the start codon, meets 9,005 bp of intronic sequence, and is spliced to an 856 bp exon 1/UTR (Fig. 1). The transcription start site (TSS, position 671,074 of supercontig 3.59) falls on the adenine nucleotide of an initiator (Inr) sequence YYANWY (Fig. 5, [50]) with a putative downstream promoter element (DPE) motif RGWY(T) at position +28. No TATA box was found. Pending functional validation, this region may thus represent a *Cxqdsx* promoter.

To qualify our *Cxqdsx* gene model, we mapped the short-read Illumina RNAseq data in NCBI SRA accession SRR991016 generated by Leal et al. [45] to supercontig 3.59 and manually annotated *Cxqdsx*. The transcript was well represented in these data, and the structure congrued with our RT-PCR and 5’RACE results in the placement and splicing of all previously described exons including the lack of additional spliceforms in female-specific exon 5 as well as the sequenced 5’ common end of exon 6. Additionally, these data allowed us to define the C-terminus of *Cxqdsx*, including the full 1,016 bp male-specific/common exon 6 and its splicing over 13,814 bp of intron to a terminal 2,201 bp 7^th^ exon/UTR (Fig. 1, Additional file 3: Figure S1). The final *Cxqdsx* protein product (Fig. 3) initiates translation in both females and males from the start codon in the common exon 2, and terminates in female mosquitoes at the opal-ochre double stop codons (conserved in Diptera, see [51]) within exon 5 and in male mosquitoes at a stop codon within exon 6. Exons 6 and 7 are thus transcribed entirely as UTR in the female isoform, as has been shown in other Dipterans including *Megaselia scalaris* [51], *Anopheles gambiae* [27] and *Aedes aegypti* [18].

**Fig. 6:**
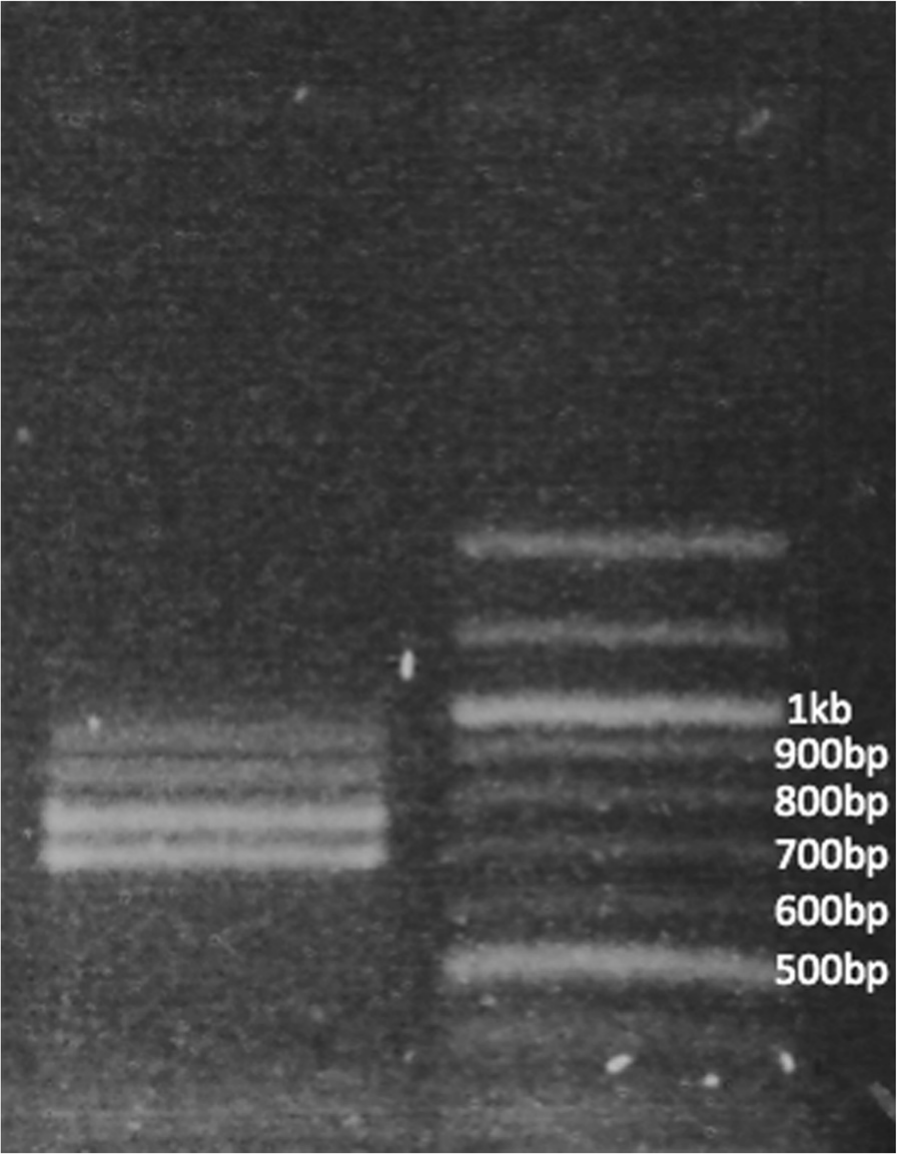
Female RT-PCR prodcuts. RT-PCR products derived from amplification of female cDNA with primers quinqOD12F/quinqOD12Rfem illustrating the four female-specific amplicons obtained by splicing of the exon 4 extension and/or the exon 2 in-frame intron (ca. 905, 830, 745 and 670 bp)

The RNAseq mapping revealed an additional alternative splicing event that was not reflected in our RT-PCR experiments; a 160 bp extension of exon 4 (exon4ex) resulting in use of an alternate downstream splice donor (Additional file 4: Figure S2) to the female-specific exon 5 acceptor. The putative peptide from this mRNA terminates within the extension at a double stop (TAATAA) codon 89 bp from the previously recognized splice donor site and encodes 30 amino acids. This is the same number of amino acids encoded by the ORF within exon 5, thus both splice forms produce peptides of equivalent length (Fig. 3). Of the 166 reads in the library splicing exons 4 and 5, 47 (28.3 %) splice exon 4 from the extension and 119 (71.7 %) from the canonical position. Our RT-PCR using male cDNA and primers quinqOD12F/DSX6R produced only the expected double-band (with and without the 75 bp in-frame intron) at 950 bp, while the female reaction using primers DSX8F (downstream of the in-frame intron) and DSX6R produced the single band mentioned previously (Fig. 4b). To address the possibility that we failed to detect a second amplicon in the latter reaction, we performed a follow-up RT-PCR on female cDNA with primers quinqOD12F/quinqOD12RF; this generated four bands (in pairs of two, Fig. 6) at sizes commensurate with those generated by removal of the exon 4 extension and/or the exon 2 in-frame intron (ca. 905, 830, 745 and 670 bp). To confirm the occurrence of the transcript variant and its restriction to female cDNA, we next searched Illumina RNAseq libraries prepared from *Cx. pipiens* f. pipiens and f. molestus, and *Cx. pipiens pallens* mosquitoes of mixed sex and life stages [46] see Additional file 2 for details and accession numbers) for presence of the exon4ex donor and for male-specific splicing of the exon 4 extension to exon 6. We found 58 amplicons (28.6 %) that spliced the extension to the female-specific exon 5 (n = 36, 13 and 9 in *Cx. p*. f. pipiens, f. molestus and *Cx. pip. pallens*, respectively) and 145 (71.4 %) from the canonical position (n = 100, 21 and 24 in *Cx. pip*. f. pipiens, f. molestus and *Cx. pip. pallens*, respectively). These numbers are nearly identical to those from *Cx. quinquefasciatus,* yet none were spliced to exon 6. This is evidence that the alternate isoform is likely specific to the female and comprises roughly 28 % of female *dsx* isoforms in the mosquitoes studied. Additionally, the alternate splice donor appears to be conserved within the *Cx. pipiens* complex. The final *Cxqdsx* gene (Fig. 1) is composed of eight exons and spans 247,017 bp of supercontig 3.59.

### Completing the *Aeadsx* gene

To compare the size, structure, intron characteristics and putative promoter regions of our full-length gene model with that of the other sequenced Culicine mosquito, *Ae. aegypti*, we used publicly available Illumina short-read RNAseq data to discern in-silico the 5’UTR, transcription start site, exon 1 and full 3’UTR of *Aeadsx* [18]. To predict the 5’ end of *Aeadsx*, we mapped Illumina short-read RNAseq libraries from NCBI SRA accession SRR789758 to *Aedes aegypti* strain Liverpool supercontig 1.370 as performed previously and located exon 2 defined by Salvemini et al. [18]. By visual inspection of the mapping, we were able to extend the 2^nd^ exon 472 bp upstream of the start codon, define a splice junction spanning 14,481 bp of intronic sequence, and locate a 1,388 bp 1^st^ exon/5’UTR (Fig. 1, Additional file 5: Figure S3). As RNAseq mapping provides only approximate definition of transcript ends, we searched for a promoter motif within an area +/− 250 bp from the point at which 5’ short-read coverage for exon 1 ceased. We located an initiator element (Inr) of the form YYANWYY at position 109460 of the reverse-complemented supercontig 1.370 and a downstream promoter element (DPE) of the form RGWYV at canonical position +28 from the Inr adenine (Fig. 5), thus providing strong evidence for the *Aeadsx* transcription start site. As in *Cxqdsx*, no TATA box was found.

These transcriptome data disagree slightly with the C-terminus of the currently described *Aeadsx* transcript (see Table 1 of Salvemini et al. [18]) in that we find 16,895 bp of intronic sequence between exon 6 and the terminal/UTR exon 7 as opposed to the 22,437 bp reported, and our data support a very large 6,382 bp 7^th^ exon (position 654172 – 660554 of reverse-complemented supercontig 3.59) as opposed to the reported 449 bp (Additional file 6: Figure S4). Additionally, we find the upstream splice acceptor to female-specific exon 5b to use canonical gt/ag splicing (Additional file 7: Figure S5) as opposed to the suboptimal gt/gt splicing reported. This does not change the comparatively high number of purines in the polypyrimidine tract or the status of exon 5b as weak (and requiring splice activation). The final *Aeadsx* gene model (Fig. 1) spanned 471,155 bp of supercontig 1.370.

**Table 1:** Splice donors and acceptors of the Cxqdsx gene. Coding (exon) sequences are in uppercase text, while the splice donor/acceptor and succeeding/preceeding 12 nucleotides, respectively, are in lowercase. “Exon 4ex” denotes the alternate downstream splice donor of exon 4. Asterisk indicates the splice acceptor site deviates significantly from the genomic mean of 8.58 +/− 1.39 SE (see Methods)

	**exon end**	**splice donor**	**intron**	**splice acceptor**	**next exon begin**	**No. purines**
exon 1	AAAAAG	gtgggcttctttatct	intron 1	ctttttcccgtttcag	ATCCTTGCTT	11
exon 2	AAGGAG	gtaagttcgcaacctc	intron 2	cctcctctctttgcag	CCAATCATGC	12
exon 3a	TACCAG	gtacgtgtcttccgct	intron 3a	cattatatcatttcag	TCCCTCCAAA	8
exon 3b	GATCAG	gtgagtgctagaagtc	intron 3b	tattatcccctttcag	ACGATGAACT	10
exon 4	ACGAAG	gtatggccgagtgttc	intron 4	ttccgttcctacgcag	GTCAAGCCGT	10
exon 4ex	TAAAAT	gtacgcaagagattcg	intron 4ex	ttccgttcctacgcag	GTCAAGCCGT	10
exon 5	TGACAG	gtacttgaactaatta	intron 5	ccaaccaacaaaacag	CTCAGGCTGT	5*
exon 6	GCGAAG	gtgagttgagcattgt	intron 6	cttatcatcattacag	ATGCCGCTAG	9
consensus		gtrnk-----------		------------ncag		

### Repetitive elements

The genera *Aedes* and *Culex* are estimated to have diverged ca. 52 Mya [44]. The genome size for *Cx. quinquefasciatus* currently stands at 540Mbp [44], while that of *Ae. aegypti* is estimated to be over twice that size at 1.3Gbp, largely due to the accumulation of transposable elements (TEs) [43]. As TEs are not distributed randomly within chromosomes [52, 53], we assessed the frequency of repetitive elements within the *doublesex* gene in order to determine whether different classes have invaded the respective *dsx* genes of *Cx. quinquefasciatus* and *Ae. aegypti*. We used CENSOR (http://www.girinst.org/censor/index.php) to scan *Cxqdsx* introns 2–7 and compared the results to those for *Aeadsx* intron 2–8 [18] (Additional file 8: Table S2, Additional file 9: Table S3). The two genes contain nearly identical numbers of DNA transposons and similar numbers of LTR retrotransposons, however *Aeadsx* was found to contain nearly twice as many Non-LTR retrotransposons (or LINEs). These elements persist with great success in eukaryote genomes [54] and comprise 4 % and 14 % of the transposable elements in the *Cx. quinquefasciatus* and *Ae. aegypti* genomes, respectively [43, 44], thus their abundance in *doublesex* likely reflects the genome-wide pattern.

### Regulatory mechanisms of *Cxqdsx*

All splice junctions of *Cxqdsx* use conserved GT-AG splice donor/acceptor motifs (Table 1). Interestingly, we find that the number of purines in the polypyrimidine tract of the 3’ splice acceptor preceding the common/male-specific exon 6 (n = 5) deviates significantly from the calculated mean (8.58, ^+^/_−_ 1.39 SE, see Methods) and constitutes a suboptimal splice acceptor. This is contrary to *Aeadsx*, which is hypothesized to activate a weak splice acceptor upstream of the female-specific exon 5b [18], and *Angdsx* which likely relies on activation of the 5’ weak splice donor downstream of exon 5 [27].

**Fig. 7:**
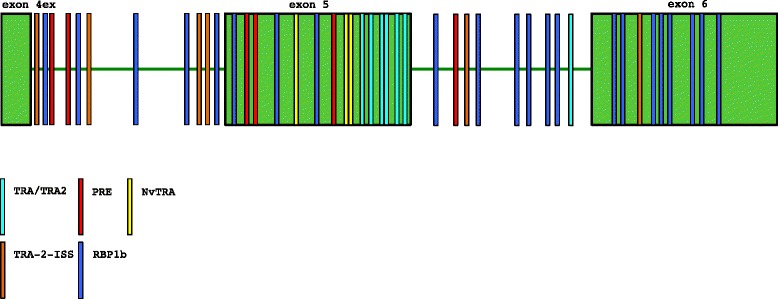
*Cis*-element distribution. Graphical representation of *cis*-element distribution within the exon 4 extension, intron 4, exon 5 (female-specific), intron 5 and exon 6 (male-specific/common UTR). Transformer/transformer 2 complex (TRA/TRA2) binding sites are colored light blue, purine-rich elements (PRE) are colored red, Nasonia-like TRA/TRA2 sites in yellow, TRA-2-ISS elements in orange and RBP2b elements in dark blue. Exons are represented as green boxes

To define putative regulatory mechanisms which may govern the sex-specific splicing of the female-specific exon 5 and/or the enhancement of the weak 3’ splice acceptor preceding the male-specific exon 6, we searched intron 4, exon 5, intron 5 and exon 6 (8,045 bp of sequence) for putative cis-acting elements derived from consensus alignments of *D. melanogaster*, *An. gambiae* (when available) and *Ae. aegypti* TRA/TRA2 binding sites (NMDNCRWNCWAYM), the *Nasonia vitripennis* TRA/TRA2 binding site (NGAAGAWN), the RBP1 type A and B motifs (DCADCTTTA and ATCYNNA) and the TRA-2-ISS motif (CAAGR, see Fig. 7 and Additional file 10: Table S4, Additional file 11: Fig. S6 for all *cis*-elements discussed below). Six copies of the TRA/TRA2 motif (two of which were overlapping) were found within a 224 bp stretch at the 3’ end of the female-specific exon 5. Three copies exhibit strong similarity (≥69 %) to the *D. melanogaster* TRA/TRA2 sequence at the nucleotide level, while the remaining three deviated from *D. melanogaster* (46-61 %) yet adhered to the consensus motif. Other Dipterans including *Drosophila* maintain six copies of the dsxRE to facilitate recruitment of splice factors to the female-specific splice site [12, 13]. Their presence may thus be evidence for a functional significance in *Cxqdsx* splicing, and the action of a TRA-like factor in splicing *Cxqdsx* pre-mRNA. To assess the significance of this cluster, we searched the *Cx. quinquefasciatus* transcriptome for additional windows of 224 bp containing six copies of the consensus motif. Two genes (.01 % of 19,019 total CDS sequences), CPIJ009301 (9 copies) and CPIJ007662 (8 copies) met this criterion. Both genes are currently annotated as 'hypothetical proteins' in VectorBase and maintain little homology to other peptides in the NCBI nr database (not shown). A single gene (CPIJ002327) contained 4 copies in 224 bp, while none remaining contained more than three. Additionally, we used the AhoPro software of Boeva et al. [47] to determine the probability of observing six copies of the motif in 8,045 bp (regardless of clustered distribution) to be 4.8 × 10^−3^. The probability of observing six copies in 224 bp is 1.91x10^−7^. Six putative purine-rich elements (PREs) were identified, three of which were in the canonical position within exon 5 near the TRA/TRA2 binding sites, however two copies were found in intron 4 and one in intron 5. The function, if any, of the latter three elements currently remains unclear. Movement of the TRA/TRA2 enhancer sites (proximal to the splice acceptor of the female-specific exon in *Drosophila dsx*) downstream to the distal splice donor of the female-specific exon (exon 5b of *Aeadsx*, see Fig. 1) appears to be conserved in the mosquitoes, however the exact effect of this placement on splicing to create the female isoform remains unknown. In *Drosophila*, they activate the splice acceptor of the female-specific exon [13], and are hypothesized to do the same to exon 5b of *Aeadsx* [18]; in *Anopheles,* they appear to activate the splice donor immediately downstream of the female-specific exon [27] (as they do in the *fruitless* gene of *D. melanogaster* [55]).

Twenty-two copies of an RBP1 type B motif were present; fourteen copies were located outside of exon 6, however these were represented by eleven different permutations of the consensus sequence. Each 7 nt permutation had a BLASTn e-value of 1.3 when queried against the full *Cxqdsx* gene sequence, and (in the absence of a clustered distribution) can be expected to occur at least once by chance. Eight copies, however, were clustered in a 546 bp stretch at the 5’ end of the male-specific exon 6. Repeating the protocol used in the TRA/TRA2-like enrichment test above, we find 86 of 19,019 transcripts (0.45 %) contain 8 or more copies of the RBP1b consensus in a 546 bp window. Many of these contigs generated positive results due to tandem repeats however (data summarized in Additional file 12: Table S5). Using AhoPro [47], we determined the probability of observing this motif in 546 bp of randomly-generated sequence data to be 2.89x10^−5^. A cluster of Rbp1 binding sites and TRA-2-ISS elements upstream of the “strong” female-specific exon 5a of *Aeadsx* are hypothesized to manage the differential splicing of this exon while other TRA/TRA2-like elements enhance the “weak” exon 5b [18] (see Fig. 1). The localization of this RBP1-binding cluster near the “weak” or suboptimal splice acceptor in *Cxqdsx* exon 6 indicates a SR-like factor may be involved in its splicing. This presents a curious model, as exon 6 is included in both male and female spliceforms. It is thus likely that if exon 6 requires activation by a SR-like factor, it would occur in the male-specific spliceform and facilitate excision of the female-specific exon 5. This would require use of the exon 6 splice acceptor at the expense of exon 5, and could be facilitated by the Rbp1 elements. The functional TRA/TRA2-like factor present in the female would then suffice to maintain incorporation of exon 6 as UTR. Five copies of the TRA-2-ISS motif were found but were not in significant representation. Three copies of the NvTRA element were found, however unlike in *Aeadsx* that maintains four copies within a cluster in exon 5, two copies were found in intron 4 and one in exon 5. The BLASTn e-value of each 8 bp hit within the search area was 0.37, thus we cannot exclude this result as having occurred simply by chance.

### Sequence evolution of *Cxqdsx*

**Fig. 8:**
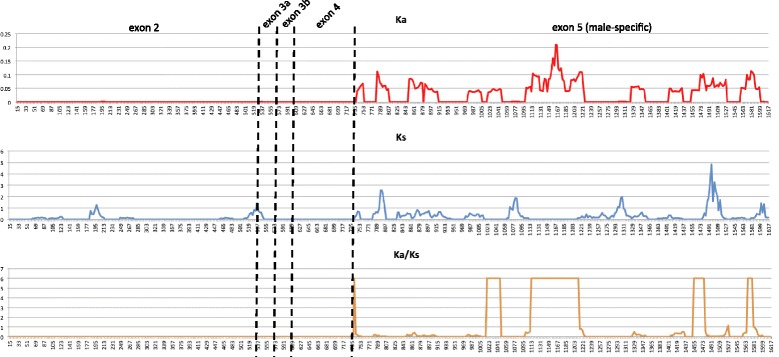
Ka/Ks graph. Graphical representation of Ka (top), Ks (middle) and Ka/Ks (bottom) values calculated for male-specific *Cx. quinquefasciatus* / *Cx. pipiens* form pipiens *doublesex* pairwise codon alignment. Values are recalculated for each 30 nucleotide (10 amino acid) window, which slides 3 nt (1 AA) at a time. Numbers on the x-axis denote the coordinate of the central nucleotide in the window. Ka/Ks values were truncated at a maximal value of six for display purposes. The common portion of the transcript ends and male-specific sequence begins with the window centered at nucleotide position 735

Assembling the complete *doublesex* transcript from two members of the *Culex pipiens* complex (*Cx. quinquefasciatus* and *Cx. pipiens* form pipiens) allowed us to examine the rate of peptide evolution within this integral gene between closely related mosquito species. Using a sliding window approach along a pairwise codon alignment of the male and female *doublesex* isoforms (Additional file 13: Figure S7, Additional file 14: Figure S8) we graphed the Ka/Ks values along the gene length. The female isoform alignment, inclusive of the common OD1 and OD2 domains, was devoid of non-synonymous substitutions and thus both Ka and Ka/Ks indicated only purifying selection. The male isoform however exhibited elevated Ka and Ks values along the majority of the male-specific C-terminus of the peptide, with ω reaching maximal values in several locations (Fig. 8, Additional file 15: Table S6). These results indicate that particular regions of the isoform may be under positive selection. The 5' end of the male-specific region has been shown to exhibit signs of positive selection in the *Anastrepha fraterculus* species group [39], however unlike *Anastrepha*, we find significantly higher levels of peptide evolution (Ka) and potential positively selected sites (Ka/Ks) in the male-specific *doublesex* transcript as compared to the female-specific and common regions in these closely related mosquitoes. Hughes [38] proposed a mechanism for this observation based on the fact that 1) *doublesex* influences not only development of insect genitalia but also of morphological and behavioral secondary sex characteristics [56–58] and 2) these secondary traits are commonly exaggerated and diverge rapidly during sexual selection in response to female choice [59]. If female choice itself were a product of neutral mutation [60], the pleiotropic repercussions of evolving linked male characters in response could create “runaway” evolutionary pressures on the male-specific DSX protein and result in the Ka and Ka/Ks patterns witnessed in our data.

## Conclusions

Our results show that the *Cx. quinquefasciatus doublesex* gene exhibits sex-specific splicing, as it does in the mosquitoes *Ae. aegypti* and *An. gambie*, as well as in other Diptera. *Cxqdsx* shares characteristics of both *Aeadsx* (gain of exon 3b, Rbp1 *cis*-regulatory binding sites) and *Angdsx* (singular female-specific exon, shared 3’ UTR), as well as a novel spliceform generated from an alternate exon 4 splice donor that appears to occur only in the female. Additionally, we complete the full-length *Aeadsx* model and identity a putative TATA-less Inr/DPE core promoter region in both *Cx. quinquefasciatus* and *Ae. aegypti* mosquito genomes, allowing for future *in situ* validation and studies of *dsx* gene transcription.

We find that *cis*-regulatory splicing regulation of *Cxqdsx* does not appear to follow either currently described mosquito model, and instead involves activation of a weak splice acceptor of the male-specific/common exon 6, possibly involving a cluster of local Rbp1 binding sites as enhancers. This finding further exemplifies the diversity present in upstream splicing regulation of *dsx* within mosquitoes, as each of the three genera studied (*Anopheles*, *Aedes* and *Culex*) possess unique regulatory mechanisms despite maintaining TRA/TRA2-like binding sites in the 3’ end of their respective female-specific exons (exon 5b in *Aeadsx*).

An analysis of peptide evolutionary rates between *Cxqdsx* and the *dsx* gene of the closely related *Cx. pipiens* form pipiens (*Cxpipdsx,* also generated in this study) shows that the male-specific component of the transcript has evolved at accelerated evolutionary rates relative to the female isoform, and contains sites exhibiting signs of positive selection. This result accentuates the rapid evolution of *doublesex* within the *Culex* species complex. Future research defining the degree to which *doublesex* influences the sexual selection cycle may shed light on the role (if any) that this integral gene plays in incipient speciation within insects.

## Availability of supporting data

The nucleotide sequences for the male and female-specific *Cxqdsx* transcripts have been submitted to GenBank under accession numbers KP033512 and KP033513, respectively. Sequences for male and female-specific *Cxpipdsx* transcripts have been submitted under accession numbers KP033514 and KP033515.

## Additional files

Additional file 1: Table S1. Primer sequences used for 5’ RACE-PCR and to amplify RT-PCR products of *Cxqdsx*. Additional file 2:
***Culex pipiens*** **NGS library preparation.** Preparation and sequencing protocol for *Culex pipiens* form pipiens RNAseq libraries. Additional file 3: Figure S1. Short-read mapping of *Cx. quinquefasciatus* RNAseq data (below; paired-end reads in blue, single-end reads in red/green) generated by Leal et al. [45] to the derived location of *Cxqdsx* exon 7 (green arrow). Reads spanning the splice junction to exon 6 are indicated with dashes at left. Data are as visualized in the CLC Genomics Workbench (CLC Bio, Aarhus, Denmark). Additional file 4: Figure S2. Short-read mapping of *Cx. quinquefasciatus* RNAseq data (below; paired-end reads in blue, single-end reads in red/green) generated by Leal et al. [45] illustrating alternate exon 4 splice donor (boxed). Reads spanning the splice junction to exon 5 are indicated with dashes. Data are as visualized in the CLC Genomics Workbench (CLC Bio, Aarhus, Denmark). Additional file 5: Figure S3. Short-read mapping of *Ae. aegypti* RNAseq data (below; paired-end reads in blue, single-end reads in red/green) from NCBI SRA accession SRR789758 illustrating the derived location of *Aeadsx* exon 1 (green arrow). Reads spanning the splice junction to exon 2 are indicated with dashes at right. The exon 1 annotation begins at the transcription start site (TSS), or the first adenine nucleotide of the initiator (Inr) sequence. Data are as visualized in the CLC Genomics Workbench (CLC Bio, Aarhus, Denmark). Additional file 6: Figure S4. Short-read mapping of *Ae. aegypti* RNAseq data (below; paired-end reads in blue, single-end reads in red/green) from NCBI SRA accession SRR789758 illustrating the derived location of *Aeadsx* exon 7 (green arrow). Reads spanning the splice junction to exon 6 are indicated with dashes at left. Data are as visualized in the CLC Genomics Workbench (CLC Bio, Aarhus, Denmark). Additional file 7: Figure S5. Splicing and alignment of RNAseq reads (numbered 1 through 14) from NCBI SRA accession SRR789758 to the *Aeadsx* exon5a/5b junction (exon 5a in yellow, 5b in green) illustrating canonical gt/ag splice donor/acceptor. Additional file 8: Table S2. CENSOR tabular output with heat-map diagram for *Cx. quinquefasciatus doublesex* introns 1 though 7. Additional file 9: Table S3. Comparison of mobile genetic elements derived from *Ae. aegypti doublesex* introns 2–8 (Salvemini et al. [15]) and *Cx. quinquefasciatus dsx* introns 2–7 (*Cxqdsx* lacks exon 5a and associated intron). Additional file 10: Table S4. Putative *cis*-element motifs of *D. melanogaster, An. gambiae, Ae. aegpyti* and *Cx. quinquefasciatus*. Genomic location of *Cx. quinquefasciatus* elements are listed. Additional file 11: Figure S6. Nucleotide sequence of exon 4 extension, intron 4, exon 5, intron 5 and exon 6 with putative *cis*-element binding sites annotated. See Fig. 6 for graphical representation. Additional file 12: Table S5. RBP1 type-b motif enrichment scan results. *Culex quinquefasciatus* transcript id, with maximum number of RBP1b motifs per 547 bp window, unique RBP1b motif sequence permutations present in the window, and nucleotide gene sequence are shown. Additional file 13: Figure S7. Amino acid (above) and nucleotide (below) aligment of *Cx. quinquefasciatus* and *Cx. pipiens* form pipiens female *doublesex* isoforms. Bolded text denotes female-specific portion of protein. Additional file 14: Figure S8. Amino acid (above) and nucleotide (below) aligment of *Cx. quinquefasciatus* and *Cx. pipiens* form pipiens male *doublesex* isoforms. Bolded text denotes male-specific portion of protein. Additional file 15: Table S6. Sliding window coordinates, Ka, Ks and Ka/Ks values calculated for each 30 bp window of *Cx. quinquefasciatus* and *Cx. pipiens* form pipiens *dsx* CDS nucleotide alignment of male isoform.
