# Supplementary material for: Characterization of the doublesex gene within the Culex pipiens complex suggests regulatory plasticity at the base of the mosquito sex determination cascade
Source: BMC Evol Biol. 2015 Jun 11;15:108. doi: 10.1186/s12862-015-0386-1 (PMC4461909; doi:10.1186/s12862-015-0386-1)
Supplement: Additional file 2: — Culex pipiens NGS library preparation. Preparation and sequencing protocol for Culex pipiens form pipiens RNAseq libraries. [file 12862_2015_386_MOESM2_ESM.docx]

**Generation of *Culex pipiens* form pipiens RNAseq library**

Egg rafts of *Cx. pipiens* f. pipiens were obtained from several water containers in the state of Baden- Württemberg in southwestern Germany. Several individual egg rafts were isolated, hatched and DNA was extracted from ca. 10 larvae from each using a Qiagen DNEasy Blood & Tissue kit (Qiagen, Valencia CA). PCR-based positive species identification of *Cx. pipiens* was performed via the acetylcholinesterase-2 assay developed by Smith and Fonseca [77], and further to f. pipiens using the CQ11 assay of Bahnck and Fonseca [19]. Thirty 1^st^/2^nd^ instar, eight 3^rd^/4^th^ instar, eight pupae and eight non-blood fed adult (4 male, 4 female) mosquitoes were isolated for extraction. Each group of specimens was placed in a separate plastic 2ml microcentrifuge tube containing a 5mm sterile stainless steel bead and 900ul QIAzol lysis reagent prior to disruption with a TissueLyser II (Qiagen, Valencia CA) for 2 minutes at 20Hz. Total RNA extraction was then carried out using the RNeasy Plus Universial kit (Qiagen, Valencia CA) per manufacturer protocol. Total RNA was quantified on a Qubit 2.0 fluorometer (Life Technologies) using the RNA Broad-range buffer. One ug of RNA from each of the four life-stage groups was combined and used to prepare an Illumina sequencing library using the TruSeq RNA Sample Prep kit v2 (Illumina, Inc. San Diego, CA) per manufacturer protocol. The library was sequenced once using 1/3 of a multiplexed 600-cycle (2x300bp paired-end) MiSeq Reagent Kit v3. Raw sequence data were quality trimmed using the CLC Genomics Workbench (Limit score cutoff = 0.05, CLC Bio, Aarhus, DK). Libraries are deposited in the NCBI Short-read Archive (SRA) under accessions SRR###### (Culex pipiens form pipiens), SRR######/SRR###### (Cx. pipiens form molestus) and SRR######/SRR###### (Cx. pipiens pallens).
