# Supplementary figures and images for: Characterization of the doublesex gene within the Culex pipiens complex suggests regulatory plasticity at the base of the mosquito sex determination cascade
Source: BMC Evol Biol. 2015 Jun 11;15:108. doi: 10.1186/s12862-015-0386-1 (PMC4461909; doi:10.1186/s12862-015-0386-1)

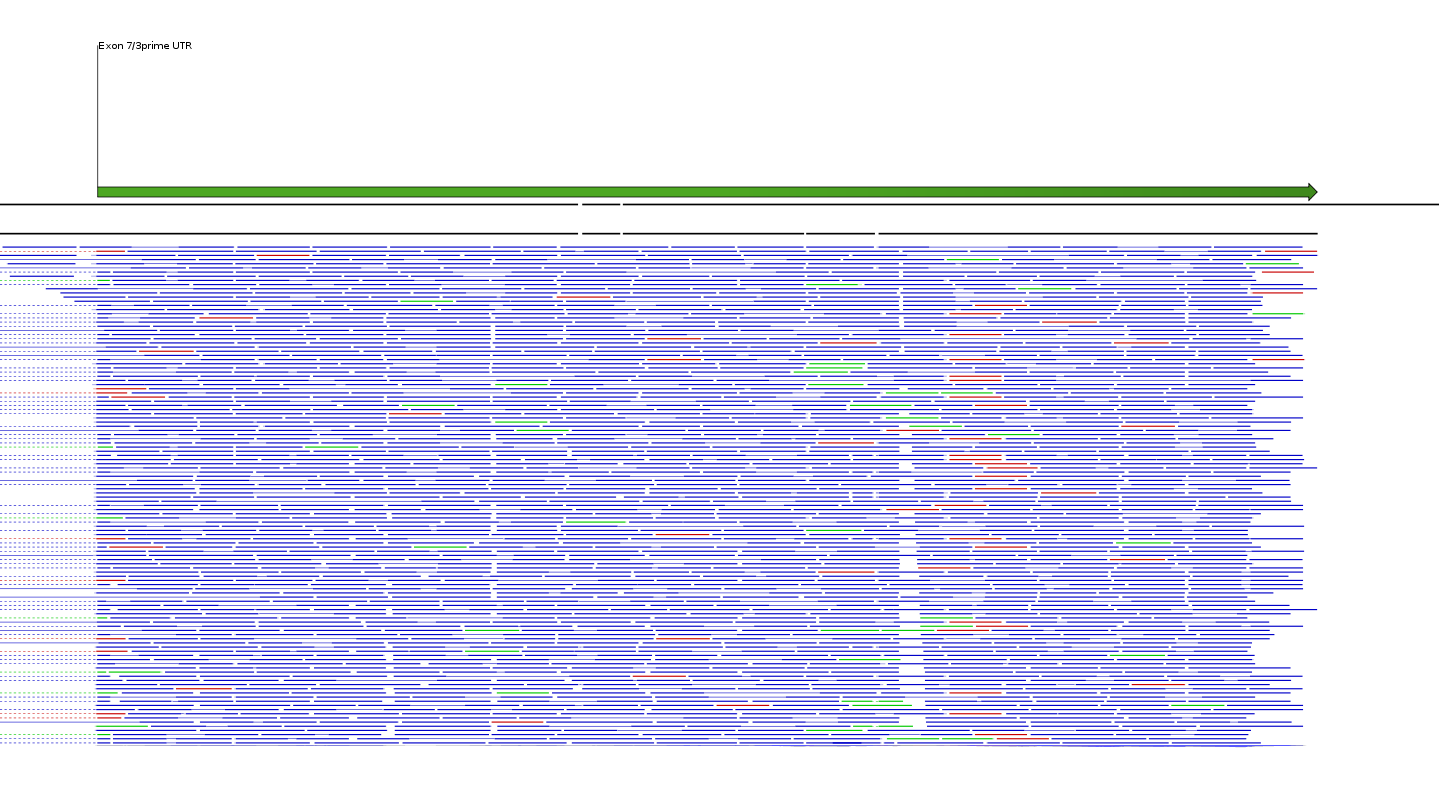

Supplement: Additional file 3: Figure S1. — Short-read mapping of Cx. quinquefasciatus RNAseq data (below; paired-end reads in blue, single-end reads in red/green) generated by Leal et al. [45] to the derived location of Cxqdsx exon 7 (green arrow). Reads spanning the splice junction to exon 6 are indicated with dashes at left. Data are as visualized in the CLC Genomics Workbench (CLC Bio, Aarhus, Denmark). [file 12862_2015_386_MOESM3_ESM.png]

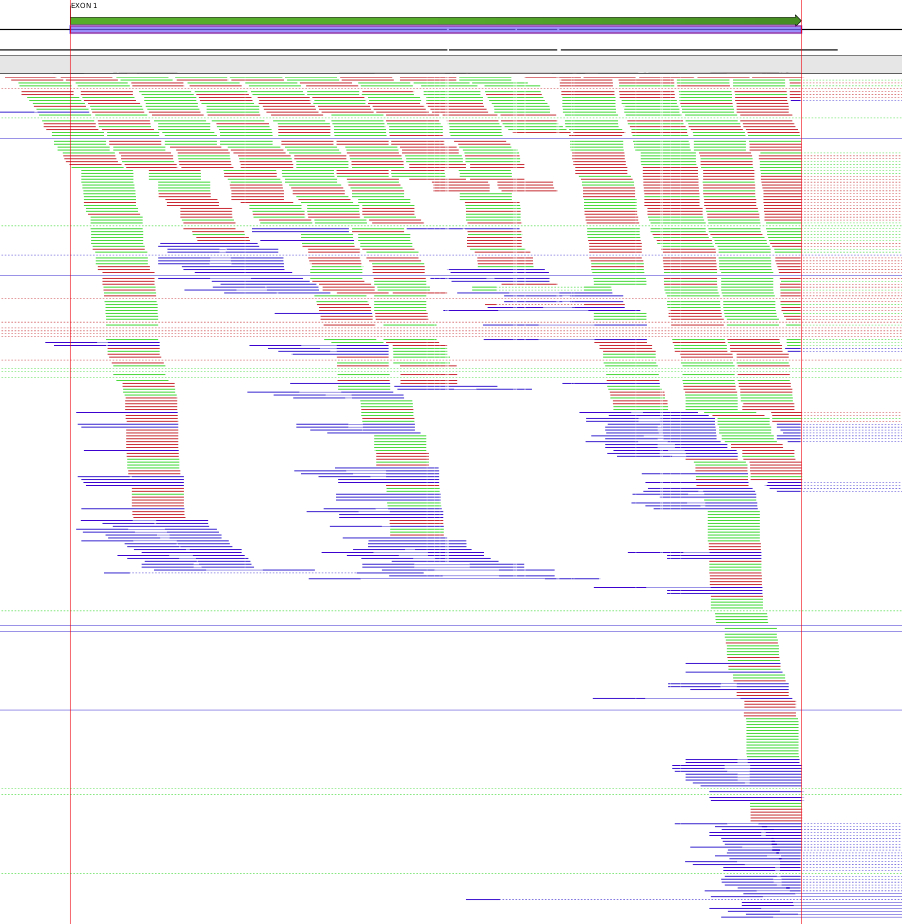

Supplement: Additional file 5: Figure S3. — Short-read mapping of Ae. aegypti RNAseq data (below; paired-end reads in blue, single-end reads in red/green) from NCBI SRA accession SRR789758 illustrating the derived location of Aeadsx exon 1 (green arrow). Reads spanning the splice junction to exon 2 are indicated with dashes at right. The exon 1 annotation begins at the transcription start site (TSS), or the first adenine nucleotide of the initiator (Inr) sequence. Data are as visualized in the CLC Genomics Workbench (CLC Bio, Aarhus, Denmark). [file 12862_2015_386_MOESM5_ESM.jpeg]

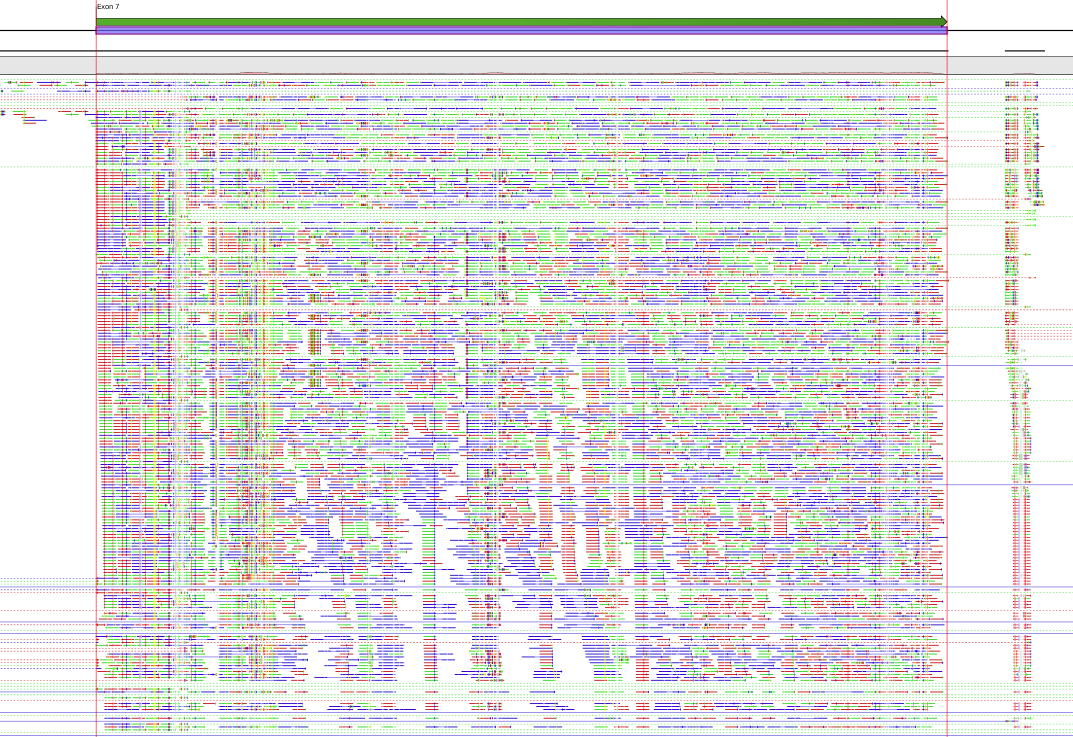

Supplement: Additional file 6: Figure S4. — Short-read mapping of Ae. aegypti RNAseq data (below; paired-end reads in blue, single-end reads in red/green) from NCBI SRA accession SRR789758 illustrating the derived location of Aeadsx exon 7 (green arrow). Reads spanning the splice junction to exon 6 are indicated with dashes at left. Data are as visualized in the CLC Genomics Workbench (CLC Bio, Aarhus, Denmark). [file 12862_2015_386_MOESM6_ESM.jpeg]
