# Supplementary material for: Characterization of the doublesex gene within the Culex pipiens complex suggests regulatory plasticity at the base of the mosquito sex determination cascade
Source: BMC Evol Biol. 2015 Jun 11;15:108. doi: 10.1186/s12862-015-0386-1 (PMC4461909; doi:10.1186/s12862-015-0386-1)
Supplement: Additional file 4: Figure S2. — Short-read mapping of Cx. quinquefasciatus RNAseq data (below; paired-end reads in blue, single-end reads in red/green) generated by Leal et al. [45] illustrating alternate exon 4 splice donor (boxed). Reads spanning the splice junction to exon 5 are indicated with dashes. Data are as visualized in the CLC Genomics Workbench (CLC Bio, Aarhus, Denmark). [file 12862_2015_386_MOESM4_ESM.pdf]

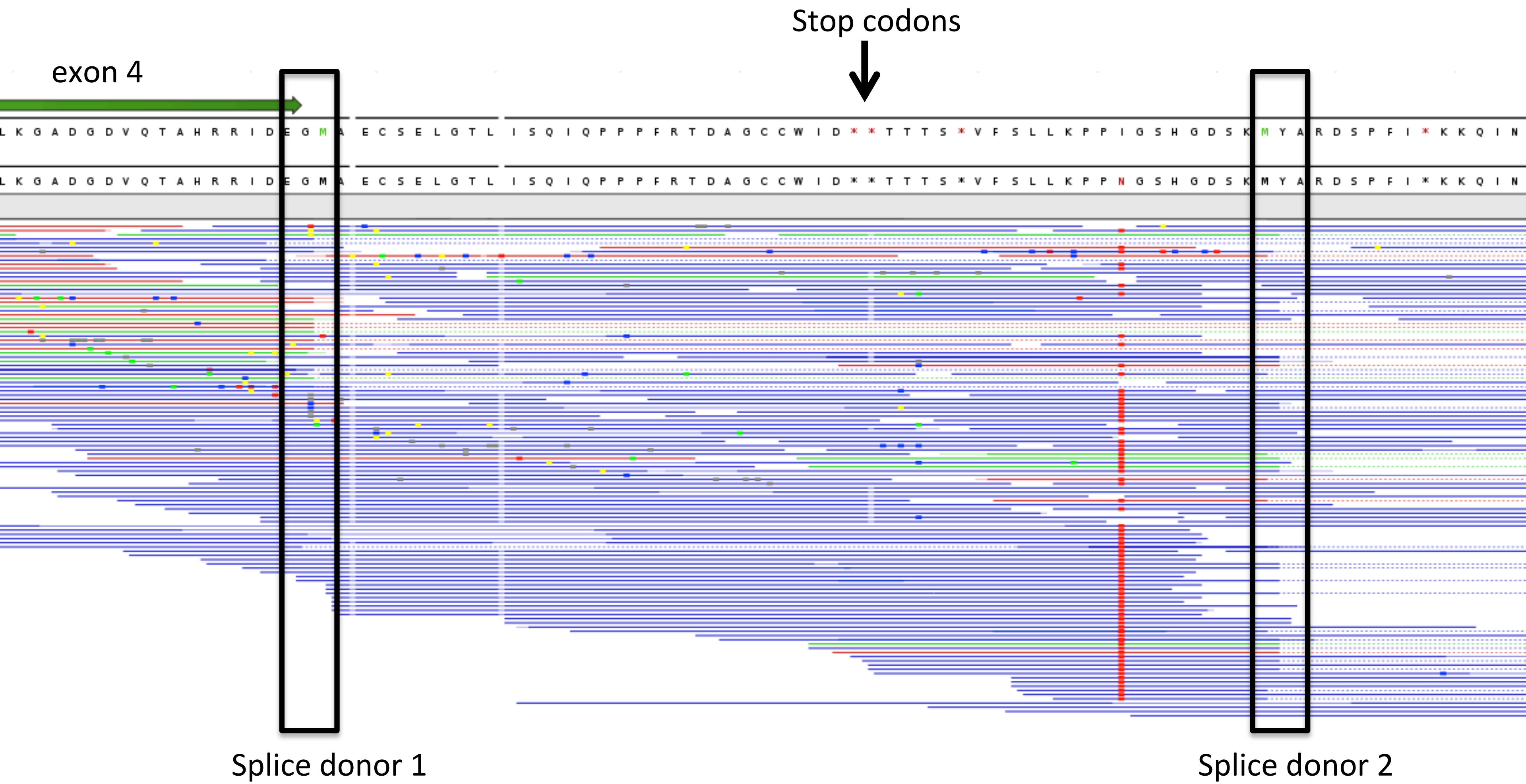
Stop codons
exon 4
Splice donor 1
Splice donor 2
