# Supplementary material for: Characterization of the doublesex gene within the Culex pipiens complex suggests regulatory plasticity at the base of the mosquito sex determination cascade
Source: BMC Evol Biol. 2015 Jun 11;15:108. doi: 10.1186/s12862-015-0386-1 (PMC4461909; doi:10.1186/s12862-015-0386-1)
Supplement: Additional file 7: Figure S5. — Splicing and alignment of RNAseq reads (numbered 1 through 14) from NCBI SRA accession SRR789758 to the Aeadsx exon5a/5b junction (exon 5a in yellow, 5b in green) illustrating canonical gt/ag splice donor/acceptor. [file 12862_2015_386_MOESM7_ESM.docx]

CONSENSUS CGTGGGTATTCGTCTGAGTTCGTCACAACTGCACCGGAAAC

1 cgtgggtattcgtctgagttcgtcacaactgcaccggaaac

2 -gtgggtattcgtctgagttcgtcacaactgcaccggaaac

3 ---gggtagtcgtctgagttcgtcacaactgcaccggaaac

4 ----------cgtctgagttcgtcacaactgcaccggaaac

5 -------------ctgagttcgtcacaactgcaccggaaac

6 -----------------------cacaactgcaccggaaac

7 ---------------------------------ccggaaac

8 -----------------------------------ggaaac

9 -------------------------------------aaac

10 -----------------------------------------

11 -----------------------------------------

12 -----------------------------------------

13 -----------------------------------------

14 -----------------------------------------

| intron 5 |

--------------------------- Exon 5a -----------------------|gtcat....cacag|-------------- Exon 5b ------------

CONSENSUS TGTGAAACAGAATAGAGCCAACCTGTGCGCGGAGAATGTTGAG| |TGCAAATGCTGTTTAACGATAATAGCGACATGCAGC

1 tgtgaaacagaatagagccaacctgtgcgcggagaatgttgag----------------tgcaaatgctgtttaac-------------------

2 tgtgaaacagaatagagccaacctgtgcgcggagaatgttgag----------------tgcaaatgctgtttaacg------------------

3 tgtgaaacagaatagagccaacctgtgcgcggagaatgttgag----------------tgcaaatgctgtttaacgat----------------

4 tgtgaaacagaatagagccaacctgtgcgcggagaatgttgag----------------tgcaaatgctgtttaacgataatagcg---------

5 tgtgaaacagaatagagccaacctgtgcgcggagaatgttgag----------------tgcaaatgctgtttaacgataatagcgaca------

6 tgtgaaacagaatagagccaacctgtgcgcggagaatgttgag----------------tgcaaatgctgtttaacgataatagcgacatgcagc

7 tgtgaaacagaatagagccaacctgtgcgcggagaatgttgag----------------tgcaaatgctgtttaacgataatagcgacatgcagc

8 tgtgaaacagaatagagccaacctgtgcgcggagaatgttgag----------------tgcaaatgctgtttaacgataatagcgacatgcagc

9 tgtgaaacagaatagagccaacctgtgcgcggagaatgttgag----------------tgcaaatgctgtttaacgataatagcgacatgcagc

10 ------acagaatagagccaacctgtgcgcggagaatgttgag----------------tgcaaatgctgtttaacgatcatagcgacatgcagc

11 ----------aatagagccaacctgtgcgcggagaatgttgag----------------tgcaaatgctgtttaacgataatagcgacatgcagc

12 ------------tagagccaacctgtgcgcggagaatgttgag----------------tgcaaatgctgtttaacgataatagcgacatgcagc

13 -----------------ccaacctgtgcgcggagaatgttgag----------------tgcaaatgctgtttaacgataatagcgacatgcagc

14 -------------------aacctgtgcgcggagaatgttgag----------------tgcaaatgctgtttaacgataatagcgacatgcagc

CONSENSUS CATTCTGGGGCTACCACGTGTAGCTCTACTTGTGAGACAGC

1 -----------------------------------------

2 -----------------------------------------

3 -----------------------------------------

4 -----------------------------------------

5 -----------------------------------------

6 catt-------------------------------------

7 cattctggggctac---------------------------

8 cattctggggctacca-------------------------

9 cattctggggctaccacg-----------------------

10 cattctggggctaccacgtgtagctctc-------------

11 cattctggggctaccacgtgtagctctacttg---------

12 cattctggggctaccacgtgtagctctacttgtg-------

13 cattctggggctaccacgtgtagctctacttgtgagaca--

14 cattctggggctaccacgtgtagctctacttgtgagacagc
