# Supplementary material for: Characterization of the doublesex gene within the Culex pipiens complex suggests regulatory plasticity at the base of the mosquito sex determination cascade
Source: BMC Evol Biol. 2015 Jun 11;15:108. doi: 10.1186/s12862-015-0386-1 (PMC4461909; doi:10.1186/s12862-015-0386-1)
Supplement: Additional file 11: Figure S6. — Nucleotide sequence of exon 4 extension, intron 4, exon 5, intron 5 and exon 6 with putative cis-element binding sites annotated. See Fig. 6 for graphical representation. [file 12862_2015_386_MOESM11_ESM.pdf]

Exon 4 alternate

GTATGGCCGAGTGTTCAGAGCTTGGGACGCTGATCAGCCAGATCCAACCGCCACCGTTCCGTACCGATGCTGGCTGTTGCTGGATTGACTAATAA

Exon 4 alternate TRA-2-ISS RBP1b 7/7 PRE

ACCACCACCTCTTGAGTTTTCAGCCTCCTCAAACCCCCAATCGGTTCTCACGGGGACAGTAAAATGTACGCAAGAGATTCGCCTTTTATCTGAAA

PRE

GAAACAAATTAATAAGGGGCGGAAAAAATGACAAGTGGGTAGGACCATCGATGTCAAGTCGCTTTCTACCAACCATGAATCGGGAGCAATTGTTTT

GGACGAGTGGAAAAGTACCTCTACACCTCAACCGAATCGGCAAAGTGCGCTGGGAATGAAAATTATGTCGTAAAATAAAAATTTACAACCATAAT

TACTCACGTCGTTCTGGCTGGAGCATGCGCCCGGCAGTCGGTGGTGGGTCAGACAACGACGGATGCGCAAACTGAACGAGATATTTCATCCGATT

GTGTGTTCGTTTGAGCAAATGTATTTTACGTAGTTATTGGGGAGCCGGAATGACCAAATCGTGCGAATGTATCATTTATATTACTAAGGGGCAAT

TATTCAATGTTGAAAGAGCCAACATTTGGTGGTGAAAGAGGTATTTTAATCACTTTTGAATATTTTTCCAATTTTTTGGCATGTTATTCTGAATT

TATTTTATAGAAGGGAATTACAAAGCATAGTTCAAATCGAAAATGTGGGTAAACGGGTTTATTTCATGGGTAATTCTCCGCCAACTCTCACAGCA

GTTGCCCCGACCCTTCTTCGATTTGCGTGAAACTTTGTCCTTAGGGGTAAATTTTGTCCCTGATCACGAACTCGAGGTCCGTTTTTTGATATCTC

PRE

GTGACGGAGGGGTGGTACGCCCCCTTCCATTTTTGAACAAGCGAAAAAAGAGGTGTTTTTCAATCATTTGCAGCCTGAAACGGTGATGTGATAGA

AATTTGGTGTCAAAGGGACTTTTATGTAAAATTGTACGCCCAATTTGATGGCGTACTCAGAATTCCGAAAAAAACATATGTTTCATCGATAAAAA

CACTTAAAAAAGTTTTAAAACTCTGCCATTTTTCGTTACTCCACTGTAAAAAATTTTGAAACTTGTCATTTTATGGGAAATTAAATGTTGTTTTC

RBP1b 7/7

GAATCTACATTGACCTAGAAGGGTCATTTTTTCAGTTAGAACAAAAAAATTCATATTAAAATTTCGTGTTTTTTCTACCTTTGCAGGATTATTTT

TAAGTGTGTAACAATGTTCGACAAAAAAAAAGTTTTAAAAAAGTTGGTCGTCGTTGATCATGACCTGGGTCACGGGATTGAAACTCGTCTGAAAAA

TRA-2-ISS

CCTGTATCATTTTCTCATTCGAAACAGTGGCGCCACGTGGTGGTAGCTGCCCTGTTTATTACACTGTGAAATTATTCATGGCCAATCCAAGGAAC

CAGAAGGTGACAACAGAAATGTCCGTCCAAAAGGGGTTCTGCAAAAAAACTTATTATTTTTAACAAATTTTCGAACAAATCAGCAACGAATTACA

AGTTTGACAGTTGAACTACACTTAAATGTTGGTTTTGTTATTTGTTTTTGCAAAACCGCATATTTTTTAACGAAAGTGCCAAAAATATTCGTAAT

CACCTTTTGCCTCTTGGTGGCGCTTTGTGTTAGTATGTGTGTGGTCGATGTTTGCGGACGTGCACGCAGAACACAGAACAGTGAGAAATAGCGAA

NvTRA/TRA2 8/8

AATGCCTCACTCCTTCTGCTACAAGTCGCCATATTGAAATTGCTTGAAGATTCATACCCGTGCCTGGGTGCTGAAAAGACAGAATGCGTAACGTG

ATTTTCGAATGCTCCCAACTGCTCCTCACTACTACAAGTGCACTACTACTGTAAACATAAACAAAGTAGAAGGCTTTATTCAAGCTCAAGCGTGA

CATATTTTTTGCTGCTGAAGTGACTGGTTTTGAAACATTTTGAATCTGTTGATGTTAAAGTTTTCGCTGTAAAATTAAGTGCTTCGTGCATTTTT

TGTTTGTAGACTAAACGATGGGCGGCCAAAATATGCTTTTTTTATTTTCCACGTGACGATAAATTGCGTCAGAAGTGGGTGGAATTTTGTGAACC

NvTRA/TRA2 6/8

AGAAGAACAACCGAATGGAAATTCCGAGATTATGTATCTTTGTATTTTAAGATTTGTTGACTTCGAATACCTCAAAAGCTGGAGTAATTTTTTTT

RBP1b 7/7

AATCCTGACAAAATAAATTATAGATCGATTACAATACTTGCCATTTCTTGGTTTCATTTTGCAAACAGTAAATTGGTTCCGCTTTGACAGTTATA

AATTGAGGCCGCTTTGCGGATAACTATTCTGCAGCCAGATCATGACCGTTCATGGTCACCCGCGACAGACACGGACGACGAAACAAAGAGAAACG

CAAAAAGTAACTTTTTCTAAACTTTATTTTTCGTAAAATCGCGATAACTTGTGACGTTTATAAGCAAACCCCTCATGTTTATATATCATTTTTTG

TAATTGTTTGCTCAACAACTTTGTAGAACATTGTTGCACTCTTAAAAATAACCCTGCAAAGTTAGAAAAAGCACAAAATTTTTAAATGAAAATTT

GTGTTCTAAATGAAAAAATTACCCTTCTAGGTCAATGTAGATTCGAAACAGAGAATTTTGAAAATTTGCCATTTTTATTTTACTAAAATGGCTGT

ATCTTGGCACTGGAGTGACAAAAACTAATTGTTAAAATCGCAATTGTTCAGAAAGAAGTTACGGTATTGGTGCCAAAAACAGTGACCTCAGGGTT

TCCGCCACATCGTTTTTTTGAGATACATCACTACGCAATCATTTTGTAAATTTTGTTAGTTCTGTGTAATTAATTATAAACAGTTGAAATAAAAT

GATAATTTTGTAATTGAGGGCTAAGATCGGCAATTTTAGGGGACTATAGTACTCATCCGGGACCGTGATGCAGGGGTAAACGTGGCTGCCTCTCA

CCCAGTCGGCTTGGGTTCGATCCCAGACGGTCCCGGTGGCATTTTTGAGACGAGATTTGTCTGACCACGCCTTCCGTTGGACGGGGAAGTAAATG

TTGGCCCCGGTCTAACCTAGAGGGTTAGGTCGTTAGCTCAATCCAGGTGTAGGAGTCGTCTCCCTGGGTCCTGCCTCGGTGGAGTCGCTGGTAGG

RBP1b 7/7

CAATTGGACTAACAATCCAAAGGTCGTCAGTTCGAATCCTGGGGTGGATGGGAAGCCTAGGTTTAAAAAGAGGTTTGCAATTGCCTCAACAATCA

TRA-2-ISS

AGCCTTCGGACAACTTAGTTTCGAGTAGGAATCTCGTAATCGAGAACGCCAAGGCAATGCTGTAGAGGGAATAATTTGATTTGATTTTTGATAGT

TRA-2-ISS RBP1b 7/7

ACTCTGGAATGGTAGATTGAACTTTTCAAACAAACTCGTCTTGCGTCCAAGGTCCAAAATCCGTAACTTGTCATTTTTCCGTCTACTGCACTACT

ACATTCGAACTGCTTAATTCCGACTATTGACCCAAATCGGAAGTGTCCCCCTGGAAAATCTCCTCCCTCTTTAGTTGAGCTCCCGTTCAACCCAT

Exon 5 RBP1b 7/7

CAAACCACCACAACCATTCGTTCTATTGTCTATTCCGTTCCTACGCAGGTCAAGCCGTCGTCAACGAATACTCACGGTTACACAATCTGAACATG

Exon 5 stop codons

TTCGACGGGGTGGAGCTTCGCAGCACCCAACGCCAGTCCGGATGATAGACTTTTTACGCAATGTCCTCCGTCGGTCCGTTGGCTCCGGCTCCACC

Exon 5 PRE PRE

GCCACTACCACCGCATCCGCTAGCCACGGACCTGAAACAGGAAAAGACGGAGCCGGGTTTACAATTTTGAAAATTGTGCGAGCGAAAAAAAAACA

PRE
Exon 5

GTGGAGATGTATGTAAAAACAAAGTTTATTGCCGAGAGCAAACAAACGAAAACCCGCAAGTTCAAACTAGTGGTTAGAGATGAACGTTGAGTTCG

Exon 5 RBP1b 7/7

ATTTTATCTAGCTGTTTCATGGAAGGTCCCTTCGATACGATCGTGGGTTTTCATCTGAAACTGGGTGGAGCAGACCGAGTGACATGCAGAACCAA

Exon 5 NvTRA/TRA2 7/8

AGGTGGTCCAAAAAAACTTGTCGTTGTCACAAATTTGTAATAATTCTTCAGGAGTGAAGAAATGCTCAATCTCTCCAACGACTTCAACCCCACTT

Exon 5 RBP1b 7/7

ACCACAAAATCTGTTCAAAACATCTCTTTTAACTATTCTCTCGATGAACTACCATAAACTTTTTGAAAACCATTCAATTCAATCCTTAAAGTGAT

Exon 5 PRE

AATGCTGTTCAGTGAACATGGCGAAATGCAGCCATTGTGGGACTACCAAGCATAGCATCTTCTGTGAGTGAGTGAAAGTGTAAAAAAAGTAAGCA

RBP1b 7/7
Exon 5 RBP1a 9/9 TRA/TRA2 9/13

TCTACGAGAAGTGCAAAGTTAAAATTTTAGGAAAGAAGTTTAACATCTTTAAGTATTAGGCAGCAAAAGTTTCGTATTGCGTGAAAAACAACCAA

TRA/TRA2 9/13 TRA/TRA2 8/13
Exon 5 TRA/TRA2 6/13 TRA/TRA2 7/13

CCTGTGTTCCGTATCCACCGATGTCGACTATTTCACCAGAATAAACCGTGCAACCAAAACATTTTCAATCAACCATGCAACCAATAATACAAAGT

Exon 5 TRA/TRA2 9/13

CAACAGCAAAACAGCCTTTTCAATCAACCATACTGTCAACCGAGGTTTTTTTTCAATCAAACAACCGGGTCAAACATTGATTGACCAAACACAGG

Exon 5 TRA/TRA2 11/13

CCATATGAATGCTTCAATCAACCAAAAAAGGGACAAATTGTTTACTCAAAATGTCCCCAAGCAGCAGGAAATGCCCTGACAGGTACTTGAACTAA

TTATATTTGGCATTGACTAATAACTGGAAAACAGACAAAAATAACAGCATGGCAATACGAACGAAAGGACATTACAGCAGGAACAATGTTTTCTT

CAATTGTTTGAACCAAACAACTTGAAGTTGTGCATTATTTTTCATAATATTCTGAATGAAAATAAACAAAAAATATTCAGCCATTGGATGGTACT

ACACAGTAAAAAATAATGTAAATTCGGAAACTGTAATTTTGGAAGGTTGAATATTACCTCTTTTATGATGTAATTTTACCTCAATTTAGACTGAA

AAAGTGACATTACACCAGAAAAGATGTAAAATTACACATTTCCAGAGGTAAAATTACACCTTTTTCTGACATAAAAGATGTACCCCTTACCATGA

TTTTTTTTCTGTGTAGCTACGAGTTTTGGCCATTCAATTTGAGAAAAAGGATTTGGGCTCCCTGGACACCCTCTAATTGACAAAATTCGATTTTA

RBP1b 7/7

GTGACTTTCCGACAATAAAAAAAATTGTAAGTTTGGATAAAAATTCAATGGAATCAATGCAACCATGCGCACCATCTTTAAAGAAACCATGAGCA

CCCCCTTATTTTACAATTGAGCAATTCTCTACCAAAACCGGAAATGGATTTTATTTGTATTTTTTGATTTGGCTCAAACTTCCCTATACCCAAAT

ATGCTATTTTGTGTCATTGGTTCACCCATACAAGTCTCCATACAATTTTGGCAGCTGTCCATACAAAAATGGTATGTAAATATTCAAACAGCTGT

PRE

AACTTTTGAGTAAATTTTTTGATAAATTTGGTGTCTTCGGCAAAGTTGTAGGTATTGTTGAGAACTTTTGAGAAAAAAAATAGGTACACGGAAAAA

PRE TRA-2-ISS

AAATTTGAGGATTTTTTATCAACTTTTTTTACTGAAACTCAATTTACCAAGATACGTATTTTTTGATTTTCGAGATTTTTTGATATGTTTTAGGG

RBP1b 7/7

GACAAAAATCCGCAACTTTTGAGCCATAGAGAAACATGGTCAAAAAATCTGCCGCCGAGTTATGAATTTTTGAAAAAATAGTGATTTTTGGAAAA

AATCGAAGTTTTATGCAAAAACAAGTTTGACATTATTTTTTAATGCAAAATTGAATTTGAAATCGAAAAGTACTTTACAGATTTTTTGATTAAGG

GCTCCGTTTTCTAGATATAGCCACCGAAAGTTTGATTTTAGCGAAATATTTGCAGTTTTTCAATTTTTAAAAATAGTGACCATGAGTAACCATTT

CTAAAAATATTTTTTTTGAAAAGTCAGAAAATTTGCTATAAAACTGTCTGAGAGACATTAAAGATTGGACCTCTTGTTGCTGAGATACAGCGGCT

TAAAGAAAAAGAAACACGAAAATTGAAGTTTTCTAAGTCTCACCCAAACAGCCCACCANNNNNNNNNNNNNNNNNNNNNNNNNNNNNNNNNNNNN

NNNNNNNNNNNNNNNNNNNNNNNNNNNNNNNNNNNNNNNNNNNNNNNNNNNNNNNNNNNNNNNNNNNNNNNNNNNNNNNNNNNNNNNNNNNNNNN

NNNNNNNNNNNNNNNNNNNNNNNNNNNNNNNNNNNNNNNNNNNNNNNNNNNNNNNNNNNNNNNNNNNNNNNNNNNNNNNNNNNNNNNNNNNNNNN

NNNNNNNNNNNNNNNNNNNNNNNNNNNNNNNNNNNNNNNNNNNNNNNNNNNNNNNNNNNNNNNNNNNNNNNNNNNNNNNNNNNNNNNNNNNNNNN

NNNNNNNNNNNNNNNNNNNNNNNNNNNNNNNNNNNNNNNNNNNNNNNNNNNNNNNNNNNNNNNNNNNNNNNNNNNNNNNNNNNNNNNNNNNNNNN

NNNNNNNNNNNNNNNNNNNNNNNNNNNNNNNNNNNNNNNNNNNNNNNNNNNNNNNNNNNNNNNNNNNNNNNNNNNNNNNNNNNNNNNNNNNNNNN

RBP1b 7/7 RBP1b 7/7

NNNNNNNNNNNNNNNNNNNNNAATATCTCGAAAATCAAAAATACGTATTTTGGGAAATTGAGTTTTAGTGAAAAAAAAGTTGATAAAAAAAATCCT

RBP1b 7/7

CATTTTTTTCCGTGTACCTATTTTTTTCTCAATAGTCCTCAACAATACCTACAACTTTGCCGAAGACACCAAATTGATCAGAACATTCACTCAAA

AGTTACAGCTGTTTGAATATTTACATACCATTTTTGTATGGACAGCAGCCAAAATTGTATGGAGACTTGTATGGGTGAACCAATGACACAAAATA

GCATATTTGGTCATAGGGAAGGCCCCCACAAAGTTTGAGTCAAATCAAAAATTACAAAAAATAAAAATGGTCGAAATCGGCCGATTTCGTAGAGA

GTTGCTCAATTTTATTTATTGGTAGGGAAACCACGAAAGTTCAATTCTGTCGATAGGAACATGTTCAGGGAGCCCACATCTAATATTTCTTAACG

AAACTGCAGCGTTTATTGTGTAATAATTTAATTGAAAAAATGGTTTGGCCACGTGTAGCGTATAAAGATCAAAAATGTTGGTTCGATGATCAAAA

RBP1b 7/7

ACTAAACTTATTTGAAATTTTCTGCAGATTTCAATTAAAATAACTGTGGAATTGTAAATTATCTTTAACATTTTTAAATGTCTCAAATAATTTGT

TTGCCTTTATATAAAATAATATGTTATTTTCAAAATTTAGTTTTAGTTTTTACTGAAAATAGCAAAAAATCAACATCGAATATCTGAGATATCGT

RBP1b 7/7

GAGTTAAAAATAAGGACATATTACCTAGGATACCTGACCAAATGACAATGAAAAATCTAAATCAACTTACAAAAAAATAAAATGAGCGAAGCAGT

TRA/TRA2 11/13

AAATATGACTTAACGAAAATCAATCAACCAGGCAATCAATGTTGACAAAATGAGTCCAACACACTGTACTATAAAATCCATCCGCTAATGTATTG

Exon 6 Male/UTR

TTTTCCACAACCCTAAATATCACAATGTCAACCCCTAAATTCCAACCAACAAAACAGCTCAGGCTGTTGTTCTGCTTCATAGCAGAATCGGTCGG

Exon 6 Male/UTR

GATGACATCGACGACGAGAACATCTCGGTGACGGGCCGTACCAACTCGACGTCCCTGTCGCGGTGTAGCTCCACGTACCGGTCGCGGTCCCGCTC

RBP1b 7/7

Exon 6 Male/UTR RBP1b 7/7

GCCACCCCATCCCGACGAGGAAGGCGTGCTCAATCTGGACACCAAGTCGGCGAAAAATGCAGCATCCGACGATTCGTCGGCGTTCAACGACGTCA

Exon 6 Male/UTR TRA-2-ISS

AGCCAAAGCAGCCAAGCGAACACCAGTCCCGGCTGGAGGAGACGTACCAGAGCAGTGTCGATCAGCACCACAGTGCCAAGTCCAAGAGTAAAAAG

Exon 6 Male/UTR

CATAGCGTTGCTGACGATGCTGAACCAGTGTCGCAAGTGGCACCTCACGAAACGAACGGCTTCGAGAAGGGTCTGAAGCTGTTCAACAATACCAA

Exon 6 Male/UTR RBP1b 7/7 RBP1b 7/7

AGCCAGCAGACGAAGCACGCACAAAGATGACTCCGTGAACGAATCTTTCGCAGCCCGTGACAAACCATTATCCTTATTTCCGAGGCATCTGCATC

RBP1b 7/7

Exon 6 Male/UTR

TAGCGGAAAATCTAGAGCTGCTCAAAACCCCGCTGTCCTTGCCATCGGCGGCAAACTTCCCACTTCCGTTCACAATTCCGCTTACCAACATGGAA

Exon 6 Male/UTR RBP1b 7/7 RBP1b 7/7 RBP1b 7/7

GCGATCCGGTCATATCCGCAGTTTTTCTATCCCTATCAAACGCACTCCGGTAGTGATCCACCCCATCCGCTGATCTCCAGTCCCTTCATGAACTA

Exon 6 Male/UTR

CCCGCACCATCCGCTGCTCTTCCCCGACGGCTACCGAAAGGAACTACCCAAGTTCCCGTGCCCCACGTCTCCGTCCAGAACGTCTTCTCCGCCGA

Exon 6 Male/UTR

AGGTGGGCGCCCAGCCTTTTTCGGGATCGCGGGTTGAACCCGTACTGCAGCCTAACCAACCGTCCGTGGCACCGATTCATTAACTGCACAATTGC

Exon 6 Male/UTR

TGAGGACCTATGAGGCGAGAGTTTGAGTTTCGAGTGAAAGGGCAGTTACACGATTGGTGATCAACCTCGACGGACTTTTCTTCCTATAGGAGGAA

Exon 6 Male/UTR

ACACCGCTTTGGAAAAGTGCTGGCGAAG
