# Supplementary material for: Characterization of the doublesex gene within the Culex pipiens complex suggests regulatory plasticity at the base of the mosquito sex determination cascade
Source: BMC Evol Biol. 2015 Jun 11;15:108. doi: 10.1186/s12862-015-0386-1 (PMC4461909; doi:10.1186/s12862-015-0386-1)
Supplement: Additional file 13: Figure S7. — Amino acid (above) and nucleotide (below) aligment of Cx. quinquefasciatus and Cx. pipiens form pipiens female doublesex isoforms. Bolded text denotes female-specific portion of protein. [file 12862_2015_386_MOESM13_ESM.docx]

**Protein**

Cxqdsx MVSQDTWMETMSESGYEGRPDGASGASSSNSLNPRTPPNCARCRNHGLKIGLKGHKRYCK

Cxpipdsx MVSQDTWMETMSESGYEGRPDGASGASSSNSLNPRTPPNCARCRNHGLKIGLKGHKRYCK

************************************************************

Cxqdsx YRSCNCEKCCLTAERQRVMALQTALRRAQTQDEQRALNDGEVAPEPVHNIHIPKLSELKE

Cxpipdsx YRSCNCEKCCLTAERQRVMALQTALRRAQTQDEQRALNDGEVAPEPVHNIHIPKLSELKE

************************************************************

Cxqdsx MKHNLMHNSQQQRSLIDCDSSTGSMNSTPGTSSMALPLHRRSPTGPVHPGEAQHLGANHA

Cxpipdsx MKHNLMHNSQQQRSLIDCDSSTGSMNSTPGTSSMALPLHRRSPTGPVHPGEAQHLGANHA

************************************************************

Cxqdsx SVSPEPANLLPVPPNIRVHHGPDSRSDDELVKRSQYLLEKLNYPWEMMPLMYVILKGADG

Cxpipdsx SVSPEPANLLPVPPNIRVHHGPDSRSDDELVKRSQYLLEKLNYPWEMMPLMYVILKGADG

************************************************************

Cxqdsx DVQTAHRRIDEGQAVVNEYSRLHNLNMFDGVELRSTQRQSG

Cxpipdsx DVQTAHRRIDEGQAVVNEYSRLHNLNMFDGVELRSTQRQSG

*****************************************

**Nucleotide**

Cxqdsx atggtttcgcaagatacctggatggagacgatgtcagaatcgggatacgaaggccggccg

Cxpipdsx atggtttcgcaagatacctggatggagacgatgtcagaatcgggatacgaaggccggccg

************************************************************

Cxqdsx gacggggccagcggtgcatccagcagtaactcgctgaacccgcggacgcccccaaactgt

Cxpipdsx gacggggccagcggtgcgtccagcagtaactcgctgaacccgcggacgcccccgaactgt

*****************.***********************************.******

Cxqdsx gcccgctgccgaaaccacgggctcaagattggcctgaagggacacaagcgttactgcaag

Cxpipdsx gcccgctgccgaaaccacgggctcaagattggcctgaagggacacaagcgttactgcaag

************************************************************

Cxqdsx tatcgcagctgcaactgcgagaaatgctgcctgacggccgaacggcagcgggtcatggcc

Cxpipdsx tatcgcagctgcaactgcgaaaaatgctgcctgacggccgaacggcagcgggtcatggcc

********************.***************************************

Cxqdsx ctgcagacggccctgcggcgggcccaaactcaggacgagcaacgagccctcaacgatggc

Cxpipdsx ctgcagacggccctgcggcgggctcaaactcaggacgagcaacgagccctcaacgatggc

***********************.************************************

Cxqdsx gaagtggcccccgaaccggtacataacattcacatacccaagctatccgaactgaaagag

Cxpipdsx gaagtggcccccgaaccggtacataacattcacatacccaagctatccgaactgaaagag

************************************************************

Cxqdsx atgaaacataatttgatgcataattctcagcagcaacgctcgttgatcgactgcgattcg

Cxpipdsx atgaaacataatttgatgcataattctcagcagcaacgctcgttgatcgactgcgattcg

************************************************************

Cxqdsx tcgaccggatcgatgaactccacaccgggcacctcgtccatggcactaccactacatcga

Cxpipdsx tcgaccggatcgatgaactccacaccgggcacctcgtccatggcactaccactgcatcga

*****************************************************.******

Cxqdsx agatcaccgacgggtccggtacatcccggcgaggcgcaacatcttggagccaatcatgcc

Cxpipdsx agatcaccgacgggtccggtacatcccggcgaggcgcaacatctcggagccaaccacgcc

********************************************.********.**.***

Cxqdsx agcgtatctcccgaacccgccaacctgttaccagtccctccaaacatcagagtacatcac

Cxpipdsx agcgtatctcccgaacccgccaacctgttaccagtccctccaaacatcagagtacatcac

************************************************************

Cxqdsx ggtccagattctcgatcagacgatgaactggtgaaacgatctcagtatctgctggagaag

Cxpipdsx ggtccagattctcgatcagacgatgaactggtgaaacgatctcagtatctgctggagaag

************************************************************

Cxqdsx ctcaactacccgtgggagatgatgcccctgatgtacgtgatactgaagggtgccgacggg

Cxpipdsx ctcaactacccgtgggagatgatgcccctgatgtacgtgatactgaagggtgccgacggg

************************************************************

Cxqdsx gacgtccaaacggcgcaccggcggatcgacgaaggtcaagccgtcgtcaacgaatactca

Cxpipdsx gacgtccaaacggcgcaccggcggatcgacgaaggtcaagccgtcgtcaacgaatactca

************************************************************

Cxqdsx cggttacacaatctgaacatgttcgacggggtggagcttcgcagcacccaacgccagtcc

Cxpipdsx cggttgcacaatctgaacatgttcgacggggtggagcttcgcagcacccaacgccagtcc

*****.******************************************************

Cxqdsx gga

Cxpipdsx gga

***
