# Supplementary material for: Characterization of the doublesex gene within the Culex pipiens complex suggests regulatory plasticity at the base of the mosquito sex determination cascade
Source: BMC Evol Biol. 2015 Jun 11;15:108. doi: 10.1186/s12862-015-0386-1 (PMC4461909; doi:10.1186/s12862-015-0386-1)
Supplement: Additional file 14: Figure S8. — Amino acid (above) and nucleotide (below) aligment of Cx. quinquefasciatus and Cx. pipiens form pipiens male doublesex isoforms. Bolded text denotes male-specific portion of protein. [file 12862_2015_386_MOESM14_ESM.docx]

**Peptide**

CxqdsxM MVSQDTWMETMSESGYEGRPDGASGASSSNSLNPRTPPNCARCRNHGLKIGLKGHKRYCK

CxpipdsxM MVSQDTWMETMSESGYEGRPDGASGASSSNSLNPRTPPNCARCRNHGLKIGLKGHKRYCK

************************************************************

CxqdsxM YRSCNCEKCCLTAERQRVMALQTALRRAQTQDEQRALNDGEVAPEPVHNIHIPKLSELKE

CxpipdsxM YRSCNCEKCCLTAERQRVMALQTALRRAQTQDEQRALNDGEVAPEPVHNIHIPKLSELKE

************************************************************

CxqdsxM MKHNLMHNSQQQRSLIDCDSSTGSMNSTPGTSSMALPLHRRSPTGPVHPGEAQHLGANHA

CxpipdsxM MKHNLMHNSQQQRSLIDCDSSTGSMNSTPGTSSMALPLHRRSPTGPVHPGEAQHLGANHA

************************************************************

CxqdsxM SVSPEPANLLPVPPNIRVHHGPDSRSDDELVKRSQYLLEKLNYPWEMMPLMYVILKGADG

CxpipdsxM SVSPEPANLLPVPPNIRVHHGPDSRSDDELVKRSQYLLEKLNYPWEMMPLMYVILKGADG

************************************************************

CxqdsxM DVQTAHRRIDE**AQAVVLLHSRIGRDDIDDENISVTGRTNSTSLSRCSSTYRSRSRSPPHP**

CxpipdsxM DVQTAHRRIDE**GQAV--LHRRIGRNDIDDENISVTGRTNSTSLSRCSSPYRSRSRSPPHL**

*************.*** ** ****:***********************.************

CxqdsxM **DEEGVLNLDTKSAKNAASDDSSAFNDVKPKQPSEHQSRLEEAYQSSVEQHHSAKSKSKKH**

CxpipdsxM **DEEGVLNLDTKSAKNAASDDSSAFNDVKPKPPSEHQSRLEEAYQNSVEQHHSAKSKSKKH**

******************************** *************.*****************

CxqdsxM **SVADDAEPVSQVAPHETNGFEKGLKLFNNTKASRRSTHKDDSVNESFAARDKPLSLFPRH**

CxpipdsxM **SVADDAEPVSQGAPHEMNGFEKGLELFKNNKANRRSTHKDDSAIESFAARDKPLSLFPRH**

************* **** *******:**:*.**.*********. ******************

CxqdsxM **LHLAENLELLKTPLSLPSAANFPLPFTIPLTNMEAIRSYPQFFYPYQTHSGSDPPHPLIS**

CxpipdsxM **LHLAENLELLKTPLSLPSAANFPLPFSIPLTNMEAIRSYPQFFYPYQTHSGSDAPHPLIS**

****************************:**************************.********

CxqdsxM **SPFMNYPHHPLLFPDGYRKELPKFPCPTSPSRTSSPPKVGAQPFSGSRVEPVLQPNQPSV**

CxpipdsxM **SPFMNYPHHSLLFPDSYRKELPKFTCPTSPSRTSSPPKVGAQPFAGSRGEPVLQPNQPSV**

***********.*****.********.*******************:*** *************

CxqdsxM **APIH**

CxpipdsxM **APIH**

********

**Nucleotide**

CxqdsxM atggtttcgcaagatacctggatggagacgatgtcagaatcgggatacgaaggccggccg

CxpipdsxM atggtttcgcaagatacctggatggagacgatgtcagaatcgggatacgaaggccggccg

************************************************************

CxqdsxM gacggggccagcggtgcatccagcagtaactcgctgaacccgcggacgcccccaaactgt

CxpipdsxM gacggggccagcggtgcgtccagcagtaactcgctgaacccgcggacgcccccgaactgt

*****************.***********************************.******

CxqdsxM gcccgctgccgaaaccacgggctcaagattggcctgaagggacacaagcgttactgcaag

CxpipdsxM gcccgctgccgaaaccacgggctcaagattggcctgaagggacacaagcgttactgcaag

************************************************************

CxqdsxM tatcgcagctgcaactgcgagaaatgctgcctgacggccgaacggcagcgggtcatggcc

CxpipdsxM tatcgcagctgcaactgcgaaaaatgctgcctgacggccgaacggcagcgggtcatggcc

********************.***************************************

CxqdsxM ctgcagacggccctgcggcgggcccaaactcaggacgagcaacgagccctcaacgatggc

CxpipdsxM ctgcagacggccctgcggcgggctcaaactcaggacgagcaacgagccctcaacgatggc

***********************.************************************

CxqdsxM gaagtggcccccgaaccggtacataacattcacatacccaagctatccgaactgaaagag

CxpipdsxM gaagtggcccccgaaccggtacataacattcacatacccaagctatccgaactgaaagag

************************************************************

CxqdsxM atgaaacataatttgatgcataattctcagcagcaacgctcgttgatcgactgcgattcg

CxpipdsxM atgaaacataatttgatgcataattctcagcagcaacgctcgttgatcgactgcgattcg

************************************************************

CxqdsxM tcgaccggatcgatgaactccacaccgggcacctcgtccatggcactaccactacatcga

CxpipdsxM tcgaccggatcgatgaactccacaccgggcacctcgtccatggcactaccactgcatcga

*****************************************************.******

CxqdsxM agatcaccgacgggtccggtacatcccggcgaggcgcaacatcttggagccaatcatgcc

CxpipdsxM agatcaccgacgggtccggtacatcccggcgaggcgcaacatctcggagccaaccacgcc

********************************************.********.**.***

CxqdsxM agcgtatctcccgaacccgccaacctgttaccagtccctccaaacatcagagtacatcac

CxpipdsxM agcgtatctcccgaacccgccaacctgttaccagtccctccaaacatcagagtacatcac

************************************************************

CxqdsxM ggtccagattctcgatcagacgatgaactggtgaaacgatctcagtatctgctggagaag

CxpipdsxM ggtccagattctcgatcagacgatgaactggtgaaacgatctcagtatctgctggagaag

************************************************************

CxqdsxM ctcaactacccgtgggagatgatgcccctgatgtacgtgatactgaagggtgccgacggg

CxpipdsxM ctcaactacccgtgggagatgatgcccctgatgtacgtgatactgaagggtgccgacggg

************************************************************

CxqdsxM gacgtccaaacggcgcaccggcggatcgacga**agctcaggctgttgttctgcttcatagc**

CxpipdsxM gacgtccaaacggcgcaccggcggatcgacga**aggtcaagccgtc------cttcaccgc**

************************************ ***.**.**. *****. ****

CxqdsxM **agaatcggtcgggatgacatcgacgacgagaacatctcggtgacgggccgtaccaactcg**

CxpipdsxM **agaatcggacggaacgacatcgacgacgagaacatctcggtgacgggccgtaccaactcg**

********** ***.*.***********************************************

CxqdsxM **acgtccctgtcgcggtgtagctccacgtaccggtcgcggtcccgctcgccaccccatccc**

CxpipdsxM **acgtccctttcgcggtgtagctccccataccggtcgcggtcccgttctccgccccatctc**

********** *************** *.*****************.** **.*******.***

CxqdsxM **gacgaggaaggcgtgctcaatctggacaccaagtcggcgaaaaatgcagcatccgacgat**

CxpipdsxM **gacgaggaaggcgttctcaatttggacaccaagtcagcgaaaaatgcagcatccgacgat**

**************** ******.*************.**************************

CxqdsxM **tcgtcggcgttcaacgacgtcaagccaaagcagccaagcgaacaccagtcccggctggag**

CxpipdsxM **tcgtcggcgttcaacgacgtcaagccaaagccaccaagcgagcaccagtcccggctggag**

********************************* .********.********************

CxqdsxM **gaggcgtaccagagcagtgtcgaacagcaccacagtgccaagtccaagagtaaaaagcat**

CxpipdsxM **gaggcgtaccagaacagtgtcgaacagcaccacagtgccaagtccaagagtaaaaagcac**

***************.*********************************************.**

CxqdsxM **agcgttgctgacgatgctgaaccagtgtcgcaagtggcacctcacgaaacgaacggcttc**

CxpipdsxM **agcgtagctgacgatgccgaaccagtgtcgcaaggggcacctcacgaaatgaacggcttc**

******* ***********.**************** **************.************

CxqdsxM **gagaagggtctgaagctgttcaacaataccaaagccagcagaagaagcacgcacaaagat**

CxpipdsxM **gagaagggtctggagctgttcaaaaataacaaagccaacagaagaagcacgcacaaagat**

**************.********** **** ********.************************

CxqdsxM **gactccgtgaacgaatctttcgcagcccgtgacaaaccattatccttatttccgaggcat**

CxpipdsxM **gactccgcgatcgaatctttcgcagcccgcgacaaaccattatccttatttccaaggcat**

*********.** ******************.***********************.********

CxqdsxM **ctgcatctagcggaaaatctagagctgctcaaaaccccgctgtccttgccatcggcggca**

CxpipdsxM **ctacatctagcggaaaatcttgagctgctcaaaaccccactctccctaccgtcagcggca**

****.***************** *****************.** ***.*.**.**.********

CxqdsxM **aacttcccacttccgttcacaattccgcttaccaacatggaagcgatccggtcatatccg**

CxpipdsxM **aacttcccacttccgttttcaattcccctcaccaacatggaagcgatccggtcatatccg**

*******************. ******* **.********************************

CxqdsxM **cagtttttctatccctatcaaacgcactccggtagtgatccaccccatccgctgatctcc**

CxpipdsxM **cagtttttctatccctaccaaacgcactccggtagtgatgctccccatccgctgatctcc**

*******************.********************* * ********************

CxqdsxM **agtcccttcatgaactacccgcaccatccgctgctcttccccgacggctaccgaaaggaa**

CxpipdsxM **agtcccttcatgaactacccgcaccattcgctgctcttccccgacagctaccggaaggaa**

*****************************.*****************.*******.********

CxqdsxM **ctacccaagttcccgtgccccacgtctccgtccagaacgtcttctccgccgaaggtgggc**

CxpipdsxM **cttcctaaattcacatgccccacgtctccgtccagaacgtcttctccgccgaaagtgggc**

**** **.**.*** *.**************************************.********

CxqdsxM **gcccagcctttttcgggatcgcgggttgaacccgtactgcagcctaaccaaccgtccgtg**

CxpipdsxM **gcccagccttttgcgggatcgcggggtgaacccgtgctgcagcccaatcaaccgtccgtg**

************** ************ *********.********.**.**************

CxqdsxM **gcaccgattcat**

CxpipdsxM **gcaccgattcat**

****************
